# Estrogen receptor-α expressing neurons in the ventrolateral VMH regulate glucose balance

**DOI:** 10.1038/s41467-020-15982-7

**Published:** 2020-05-01

**Authors:** Yanlin He, Pingwen Xu, Chunmei Wang, Yan Xia, Meng Yu, Yongjie Yang, Kaifan Yu, Xing Cai, Na Qu, Kenji Saito, Julia Wang, Ilirjana Hyseni, Matthew Robertson, Badrajee Piyarathna, Min Gao, Sohaib A. Khan, Feng Liu, Rui Chen, Cristian Coarfa, Zhongming Zhao, Qingchun Tong, Zheng Sun, Yong Xu

**Affiliations:** 1 Children’s Nutrition Research Center, Department of Pediatrics, Baylor College of Medicine, Houston, TX 77030 USA; 2 Department of Molecular and Cellular Biology, Baylor College of Medicine, Houston, TX 77030 USA; 3 Department of Molecular and Human Genetics, Baylor College of Medicine, Houston, TX 77030 USA; 4 Department of Cell and Cancer Biology, Vontz Center for Molecular Studies, University of Cincinnati, College of Medicine, Cincinnati, OH 45267 USA; 5 Departments of Pharmacology, University of Texas Health at San Antonio, San Antonio, TX 78229 USA; 6 Center for Precision Health, School of Biomedical Informatics, The University of Texas Health Science Center at Houston, Houston, TX 77030 USA; 7 Brown Foundation Institute of Molecular Medicine, University of Texas Health Science Center at Houston, Houston, TX 77030 USA; 8 Department of Medicine, Division of Diabetes, Endocrinology and Metabolism, Baylor College of Medicine, Houston, TX 77030 USA

**Keywords:** Neuroscience, Physiology, Endocrinology

## Abstract

Brain glucose-sensing neurons detect glucose fluctuations and prevent severe hypoglycemia, but mechanisms mediating functions of these glucose-sensing neurons are unclear. Here we report that estrogen receptor-α (ERα)-expressing neurons in the ventrolateral subdivision of the ventromedial hypothalamic nucleus (vlVMH) can sense glucose fluctuations, being glucose-inhibited neurons (GI-ERα^vlVMH^) or glucose-excited neurons (GE-ERα^vlVMH^). Hypoglycemia activates GI-ERα^vlVMH^ neurons via the anoctamin 4 channel, and inhibits GE-ERα^vlVMH^ neurons through opening the ATP-sensitive potassium channel. Further, we show that GI-ERα^vlVMH^ neurons preferentially project to the medioposterior arcuate nucleus of the hypothalamus (mpARH) and GE-ERα^vlVMH^ neurons preferentially project to the dorsal Raphe nuclei (DRN). Activation of ERα^vlVMH^ to mpARH circuit and inhibition of ERα^vlVMH^ to DRN circuit both increase blood glucose. Thus, our results indicate that ERα^vlVMH^ neurons detect glucose fluctuations and prevent severe hypoglycemia in mice.

## Abstract (web summary)

Glucose-sensing neurons are found in the ventromedial hypothalamic nucleus (VMH). Here the authors identify the role of estrogen receptor-α expressing neurons in the ventrolateral subdivision of the VMH in sensing hypoglycemia.

## Introduction

Severe hypoglycemia is a life-threatening problem for diabetic patients with intensive insulin therapy^1^. While normal subjects can correct isolated hypoglycemic events through a defending mechanism, this protection is often impaired in diabetic patients. Elimination of hypoglycemia from the lives of diabetic patients and long-term maintenance of euglycemia will require critical fundamental insights into the mechanisms for defending against hypoglycemia.

Although all neurons need glucose as a basic fuel for neuronal viability and functions, not all neurons rapidly change their firing activity and membrane potential in response to glucose fluctuations, a feature named glucose sensing. Glucose-sensing neurons are found in several brain regions, including the ventromedial hypothalamic nucleus (VMH, also known as VMN), the arcuate nucleus (ARH), the paraventricular nucleus of the hypothalamus (PVH), the nucleus of solitary tract (NTS), and the medial amygdala^2–5^. In particular, the VMH has been well-documented in the regulation of glucose balance^6^. Many VMH neurons are glucose sensing, being excited by high glucose level (glucose excited, GE) or being inhibited by high glucose (glucose inhibited, GI)^7^. Local glucopenia produced by infusion of 2-deoxy-D-glucose (2-DG, a glucose metabolism antagonist) into rat VMH significantly increases glucagon levels in the circulation, associated with elevated blood glucose^8^; whereas infusions of glucose directly into the VMH blocks glucagon release despite of the systemic hypoglycemia^9^. Mice with genetic loss of glutamatergic neurotransmission only in VMH neurons display impaired responses to hypoglycemia^10^. UCP2-dependent mitochondrial fission in VMH neurons have been recently reported to mediate glucose-induced neuronal activation and therefore regulate the whole-body glucose metabolism^11^. Abundant neurons in the dorsomedial subdivision of the VMH (dmVMH) express glucokinase, and activation of these neurons increase blood glucose in mice^12^; deletion of glucokinase reduces hypoglycemia-induced glucagon secretion^13^. Further, neurons in the dmVMH and those in the central subdivision of VMH (cVMH) receive neuronal inputs from glucose-sensing neurons in the parabrachial nucleus (PBN) to defend against hypoglycemia^14,15^. Together, these findings strongly support an essential role of dmVMH/cVMH neurons in the regulation of glucose balance. However, neurons in another VMH subdivision, the ventrolateral VMH (vlVMH), also respond to glucose fluctuations^11^, but their functions in glucose metabolism have not been specifically investigated.

Estrogen receptor-α (ERα) is abundantly expressed in the vlVMH, but largely spared in the dmVMH and the cVMH^16^. In the present study, we systematically characterized the glucose-sensing properties of these ERα-expressing neurons in the vlVMH (ERα^vlVMH^ neurons). We used fiber photometry, optogenetics, and CRISPR-Cas9 approaches to identify the ionic and circuitry mechanisms by which these neurons sense glucose fluctuations and regulate blood glucose levels.

## Results

### All tested ERα^vlVMH^ neurons sense glucose fluctuations

**Fig. 1:**
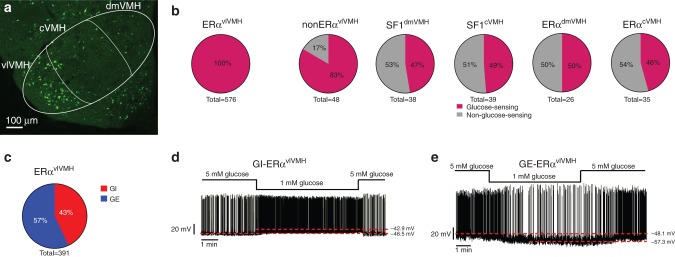
ERα^vlVMH^ neurons are glucose sensing. **a** Direct visualization of ZsGreen-labeled ERα neurons in a coronal hypothalamic section from a female ERα-ZsGreen mouse. The VMH subdivisions, dmVMH, cVMH, and vlVMH, were highlighted with white borders. The similar results were replicated three times. **b** Percentage of glucose-sensing or non-glucose-sensing neurons in different VMH subgroups. **c** Percentage of GI and GE neurons within the ERα^vlVMH^ population in gonad-intact female mice. **d**, **e** Representative electrophysiological responses to glucose fluctuations (5→1→5 mM) in a GI **d** and a GE-ERα^vlVMH^ neuron **e**. Source data are provided as a Source Data Fig. 1.

We have recently generated and validated a new ERα-ZsGreen mouse strain, in which expression of a fluroscence reporter, ZsGreen, is driven by the mouse ERα promoter. As we reported^17^, ZsGreen is selectively expressed in ERα-expressing neurons, including those in the vlVMH (Fig. 1a). We used female ERα-ZsGreen mice to record glucose-sensing properties of ERα^vlVMH^ neurons under the current clamp mode in response to a 5→1→5 mM extracellular glucose fluctuation protocol^18^. Strikingly, all the tested ERα^vlVMH^ neurons (576 neurons from 65 mice) are glucose-sensing neurons (defined as >2 mV depolarization or hyperpolarization in response to the glucose fluctuations)^19^. We also examined the adjacent non-ERα^vlVMH^ neurons (ZsGreen(−) neurons) and found that 83% of these neurons are glucose sensing, while the rest 17% non-ERα^vlVMH^ neurons did not respond to glucose fluctuations (Fig. 1b; *P* < 0.0001 compared to ERα^vlVMH^ neurons, *χ*^2^ test). Interestingly, only 47% of dmVMH neurons and 49% of cVMH neurons (labeled by the SF1 promoter) are found to be glucose sensing, using the same recording protocol (Fig. 1b; *P* < 0.0001 compared to ERα^vlVMH^ neurons, *χ*^2^ tests). Since scattered ERα neurons are also present in the dmVMH and cVMH (Fig. 1a), we examined the glucose-sensing properties of these neurons, and found only 50% of ERα^dmVMH^ neurons and 46% of ERα^cVMH^ neurons responded to glucose fluctuations (Fig. 1b; *P* < 0.0001 compared to ERα^vlVMH^ neurons, *χ*^2^ tests). Thus, ERα^vlVMH^ neurons represent a unique subpopulation with remarkably strong glucose-sensing capability.

Among ERα^vlVMH^ neurons, 43% of them depolarized and increased their firing frequency in response to hypoglycemia (5→1 mM glucose), while recovery of glucose level to 5 mM restored activities of all these neurons (Fig. 1c, d); these neurons were identified as GI-ERα^vlVMH^ neurons. The rest 57% of female ERα^vlVMH^ neurons hyperpolarized and decreased their firing frequency in response to hypoglycemia, and then recovered at 5 mM glucose condition (Fig. 1c, e); these neurons were identified as GE-ERα^vlVMH^ neurons. We exposed female ERα^vlVMH^ neurons to 5→2.5→1→2.5→5 mM glucose fluctuation, and found that GE- and GI-ERα^vlVMH^ neurons changed their membrane potential in a concentration-dependent manner (Supplementary Fig. 1a, b). We repeated the same 5→1→5 mM extracellular glucose fluctuation protocol in the presence of a cocktail of synaptic blockers (TTX, CNQX, D-AP5, and bicuculline) and found similar hypoglycemia-induced depolarization in GI-ERα^vlVMH^ neurons and hyperpolarization in GE-ERα^vlVMH^ neurons (Supplementary Fig. 1c–d), indicating that glucose-sensing of ERα^vlVMH^ neurons is independent of synaptic inputs.

### GI- and GE-ERα^vlVMH^ neurons use distinct ionic conductances

**Fig. 2:**
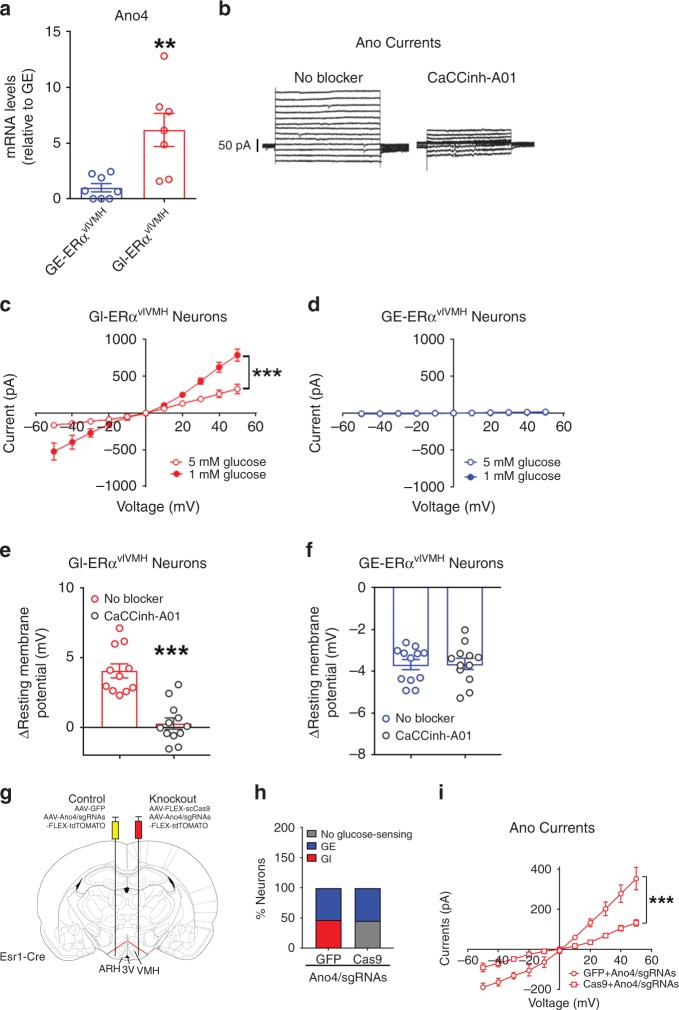
Ano4 mediates hypoglycemia-induced activation in GI-ERα^vlVMH^ neurons. **a** Relative mRNA levels of Ano4 in female GI-ERα^vlVMH^ neurons and GE-ERα^vlVMH^ neurons measured by real-time RT-qPCR. *N* = 7 or 8 neurons from three mice per group. Results are shown as mean ± SEM. **P = 0.0033 in two-sided *t*-tests. **b** Typical Ano current in a female GI-ERα^vlVMH^ neuron in the presence or absence of an Ano inhibitor (100 μM CaCCinh-A01). **c**, **d** Ano currents measured in female GI-ERα^vlVMH^ neurons (**c**, *N* = 10 or 17 neurons from three mice per group) and GI-ERα^vlVMH^ neurons (**d**, *N* = 5 or 9 neurons from two mice per group) in the presence of high (5 mM) or low (1 mM) glucose. Results are shown as mean ± SEM. ***P < 0.0001 in two-way ANOVA repeated measurements followed by Sidak post hoc tests. **e**, **f** Hypoglycemia-induced changes in resting membrane potential in female GI-ERα^vlVMH^ neurons **e** and in GE-ERα^vlVMH^ neurons **f** in the absence or the presence of 100 μM CaCCinh-A01. *N* = 11 or 12 neurons from three mice per group. Results are shown as mean ± SEM. ***P < 0.0001 in two-sided *t*-tests. **g** Schematic representation of CRISPR-mediated knockout of Ano4 in one side of ERα^vlVMH^ neurons but leaving the other side as controls in the same female mice. **h** The composition (%) of GE, GI, and no glucose-sensing neurons from ERα^vlVMH^ populations in the control and Ano4-knockout sides. *N* = 19 or 24 neurons from two mice per group. **i** Ano currents measured in the control and Ano4-knockout sides of female ERα^vlVMH^ neurons in the presence of high (5 mM) glucose. Note: since there were no GI neurons in the knockout side, data from Ano4-knockout side were collected from nonGE neurons **h**, while data for control side were collected from identified GI neurons. *N* = 7 or 8 neurons from two mice per group. ***P = 0.0002 in two-way ANOVA repeated measurements followed by Sidak post hoc tests. Source data are provided as a Source Data Fig. 2.

We used the Patch-seq approach^20^ to further explore the mechanisms by which female GI- and GE-ERα^vlVMH^ neurons sense and respond to glucose fluctuations. We first used patch-clamp recordings to identify single GI- and GE-ERα^vlVMH^ neurons from ERα-ZsGreen female mice, and then manually collected these neurons for transcriptome experiments (RNA-seq). Our analysis revealed 372 genes differentially expressed in GE- vs. GI-ERα^vlVMH^ neurons (*P* < 0.05 and |log_2_(fold change)| > 2; Supplementary Tables 1 and 2, Supplementary Data 1). Our functional enrichment analysis revealed 23 Gene Ontology (GO) terms that are statistically enriched in these genes (Supplementary Data 2). Among them, “ATP binding” (GO:0005524) is the second most enriched GO Molecular Function term (Supplementary Data 2). Other GO terms included “regulation of response to external stimulus” (GO:0032101), “plasma membrane region” (GO:0098590), and “transporter activity” (GO:0005215; Supplementary Data 2). These results suggested that differentially expressed genes involved in ATP binding, transporter, and membrane regions may account for the opposite electrophysiological responses of GI- and GE-ERα^vlVMH^ to glucose fluctuations. Among these differently expressed genes, we paid attention to those known to encode ion channels as potential targets. In particular, expression of anoctamin 4 gene (*Ano4*, encoding a calcium-activated chloride channel protein) was significantly higher in GI-ERα^vlVMH^ neurons than in GE-ERα^vlVMH^ neurons (*P* = 0.0133, log_2_(fold change) = 3.102, Supplementary Fig. 2a). Our qPCR assay further confirmed that expression of *Ano4* is significantly higher in GI-ERα^vlVMH^ neurons than GE-ERα^vlVMH^ neurons (Fig. 2a, primer sequences seen in Supplementary Table 3). Consistently, we detected robust rectifying currents in GI-ERα^vlVMH^ neurons that were blocked by CaCCinh-A01 (1 µM), an anoctamin inhibitor^21^, confirming that these were Ano currents (Fig. 2b). Importantly, these Ano currents in GI-ERα^vlVMH^ neurons were significantly potentiated by exposure to low glucose (1 mM) compared to high glucose (5 mM), whereas such currents were minimal in GE-ERα^vlVMH^ neurons regardless of glucose concentrations (Fig. 2c, d). Further, CaCCinh-A01 abolished the responsiveness of GI-ERα^vlVMH^ neurons to glucose fluctuations, but it had no effect on GE-ERα^vlVMH^ neurons (Fig. 2e, f). To further confirm the role of Ano4, we used CRISPR-Cas9 approach to knockout *Ano4* specifically in ERα^vlVMH^ neurons. Briefly, we designed sgRNAs targeting exon 4 and exon 11 of the *Ano4* gene, respectively, screened 19 sgRNAs, and identified two sgRNAs that effectively induced indel mutations in each exon in the HEK293 cells (Supplementary Fig. 2b). These two sgRNAs were constructed into an AAV vector followed by Cre-dependent FLEX-tdTOMATO sequence (Supplementary Fig. 2c). Female Esr1-Cre mice received stereotaxic injections of AAV-FLEX-scCas9 (Vector Biolabs, #7122) and AAV-Ano4/sgRNAs-FLEX-tdTOMATO into one side of the vlVMH to disrupt expression of *Ano4* selectively in ERα^vlVMH^ neurons. For the purpose of the control, the other side of the vlVMH received AAV-Ano4/sgRNAs-FLEX-tdTOMATO and the AAV-GFP (no Cas9) virus (Fig. 2g). Compared to control side (GFP + Ano4/sgRNA), the combination of Cas9 and Ano4/sgRNA diminished the GI population without affecting the GE population, and robustly reduced Ano currents in TOMATO-labeled ERα^vlVMH^ neurons that were not GE (Fig. 2h, i). Thus, our results indicate that *Ano4* is required for GI-ERα^vlVMH^ neurons to respond to glucose fluctuations.

**Fig. 3:**
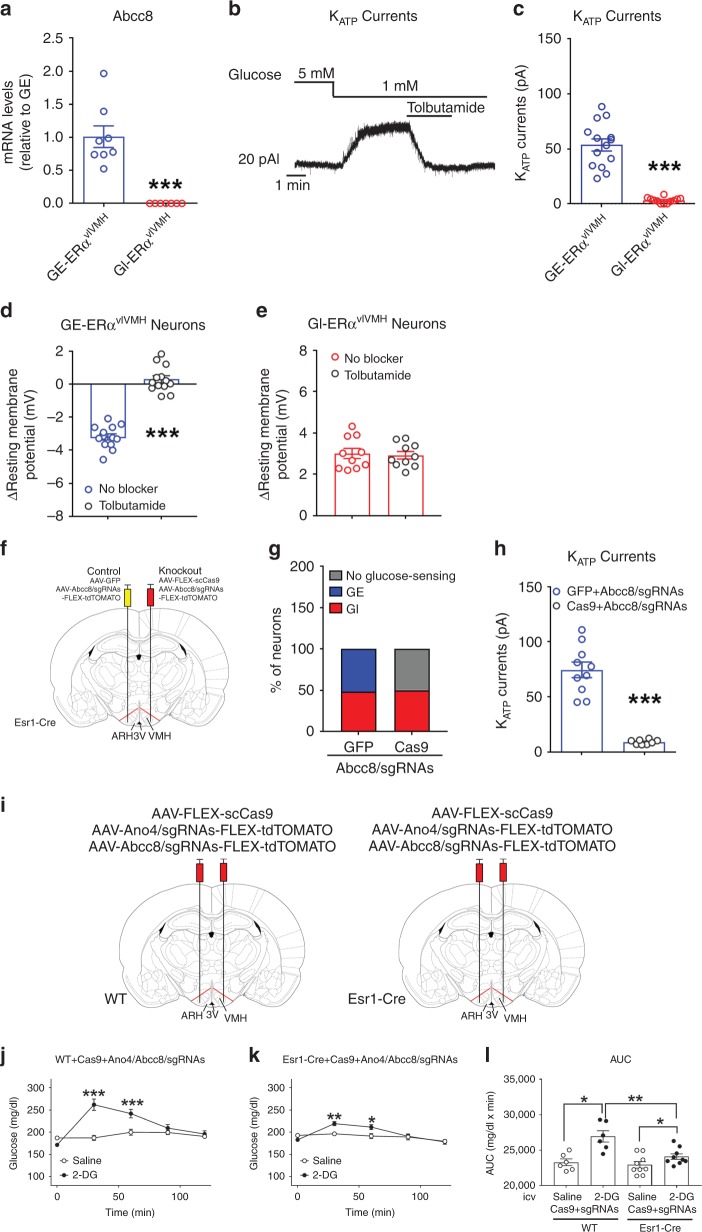
Abcc8 mediates hypoglycemia-induced inhibition GE-ERα^vlVMH^ neurons. **a** Relative mRNA levels of Abcc8 in female GI- and GE-ERα^vlVMH^ neurons measured by real-time RT-qPCR. *N* = 7 or 8 neurons from three mice per group. ***P < 0.0001 in two-sided *t*-tests. **b** K_ATP_ current in a female GE-ERα^vlVMH^ neuron in response to glucose fluctuations with or without tolbutamide (200 µM). **c** Hypoglycemia-induced K_ATP_ currents in female GE- and GI-ERα^vlVMH^ neurons. *N* = 12 or 14 neurons from three mice per group. ***P < 0.0001 in two-sided *t*-tests. **d**, **e** Hypoglycemia-induced changes in resting membrane potential in female GE- **d** or GI-ERα^vlVMH^ neurons **e** in the absence or the presence of tolbutamide (200 µM). *N* = 10 or 13 neurons from three mice per group. ***P < 0.0001 in two-sided *t*-test. **f** CRISPR-mediated knockout of Abcc8 in one side of ERα^vlVMH^ neurons leaving the other side as controls in the same female mice. **g** The composition (%) of GE, GI, and no glucose-sensing neurons from ERα^vlVMH^ populations in the control and Abcc8-knockout sides. *N* = 19 or 20 neurons from two mice per group. **h** K_ATP_ currents measured in the control and Abcc8-knockout sides of female ERα^vlVMH^ neurons in the presence of 1 mM glucose. Note: since there were no GE neurons in the knockout side, data from knockout side were collected from nonGI neurons **g**, while data for control side were collected from identified GE neurons. *N* = 8 or 10 neurons from two mice per group. ***P < 0.0001 in two-sided *t*-tests. **i** CRISPR-mediated knockout of both Ano4 and Abcc8 in both sides of ERα^vlVMH^ neurons in Esr1-Cre female mice, while WT mice were used as controls. **j**–**l** Effects of icv saline or 2-DG on blood glucose in control **j** or in mice with both Ano4 and Abcc8 disrupted in ERα^vlVMH^ neurons **k**. *N* = 6 or 9 mice per group. *P = 0.0185, **P = 0.0053, at 30 min ***P < 0.0001 and at 60 min ***P = 0.0007 in two-way ANOVA analysis followed by post hoc Sidak tests. **l** Area under the curve for data in **j** and **k**. *N* = 6 or 9 mice per group. In WT *P = 0.0139, in Esr1-Cre *P = 0.0104, and **P = 0.0036 in two-sided *t*-tests. Results in **a**, **c**–**e**, **h**, **j**–**l** are shown as mean ± SEM. Source data are provided as a Source Data Fig. 3.

The Patch-seq analysis also revealed that expression of *Abcc8* (which encodes the Sur1 protein, one subunit of the K_ATP_ channel) was substantially higher in GE-ERα^vlVMH^ neurons than that in GI-ERα^vlVMH^ neurons (*P* = 0.0088, log_2_(fold change) = 4.597, Supplementary Fig. 2a). Our qPCR analyses further confirmed that *Abcc8* mRNAs were abundant in GE-ERα^vlVMH^ neurons but below the detection threshold in GI-ERα^vlVMH^ neurons (Fig. 3a, primer sequences seen in Supplementary Table 3). Consistently, we showed that K_ATP_ channel-mediated outward currents in female GE-ERα^vlVMH^ neurons were significantly elevated by hypoglycemia, which were blocked by 200 µM tolbutamide, a K_ATP_ channel inhibitor^18^ (Fig. 3b). On the other hand, such K_ATP_ channel-mediated outward currents were almost not detectable in female GI-ERα^vlVMH^ neurons (Fig. 3c). In addition, treatment of tolbutamide (200 µM) blocked hypoglycemia-induced inhibition in female GE-ERα^vlVMH^ neurons but had no effect on GI-ERα^vlVMH^ neurons (Fig. 3d, e). To further confirm the function of Abcc8, we designed and identified two sgRNAs that efficiently induced indel mutations in exon 2 and exon 5 of the *Abcc8* gene (Supplementary Fig. 2d). Both these sgRNAs were constructed into one AAV vector followed by Cre-dependent FLEX-tdTOMATO sequence (AAV-Abcc8/sgRNAs-FLEX-tdTOMATO; Supplementary Fig. 2e). Female Esr1-Cre mice received stereotaxic injections of AAV-FLEX-scCas9 and AAV-Abcc8/sgRNAs-FLEX-tdTOMATO into one side of the vlVMH. As controls, the other side of vlVMH of the same mice received AAV-Abcc8/sgRNAs-FLEX-tdTOMATO and AAV-GFP (no Cas9; Fig. 3f). Compared to the control side (GFP + Abcc8/sgRNA), the combination of Cas9 and Abcc8/sgRNA diminished the GE population without affecting the GI population and robustly reduced K_ATP_ currents in TOMATO-labeled ERα^vlVMH^ neurons that were not GI (Fig. 3g, h). Thus, our results indicate that hypoglycemia opens the K_ATP_ channel in female GE-ERα^vlVMH^ neurons to inhibit these neurons.

We further examined the functions of Ano4 and Abcc8 and in ERα^vlVMH^ neurons in the regulation of glucose balance in vivo using intracerebroventricular (icv) injections of 2-DG to induce central glucopenia^10^. In control female mice (wild-type mice receiving stereotaxic injections of AAV vectors that express scCas9, Ano4/sgRNAs, and Abcc8/sgRNAs into two sides of the vlVMH; Fig. 3i), icv 2-DG significantly elevated blood glucose compared to icv saline in the same mice (Fig. 3j). On the other hand, the 2-DG-induced glucose elevations were largely attenuated in female mice with simultaneous disruption of Ano4 and Abcc8 in ERα^vlVMH^ neurons (female Esr1-Cre mice receiving stereotaxic injections of AAV vectors that express scCas9, Ano4/sgRNAs, and Abcc8/sgRNAs into two sides of the vlVMH; Fig. 3i–l). Together, these results indicate that Ano4 and Abcc8 mediate glucose-sensing functions of GI- and GE-ERα^vlVMH^ neurons, respectively, and their functions are required for normal glucose balance in female mice. It is worth noting that this study did not directly address the downstream hormonal and/or neural signals mediating the regulations on glucose, which warrants future investigations.

### GI- and GE-ERα^vlVMH^ neurons recruit different circuits

**Fig. 4:**
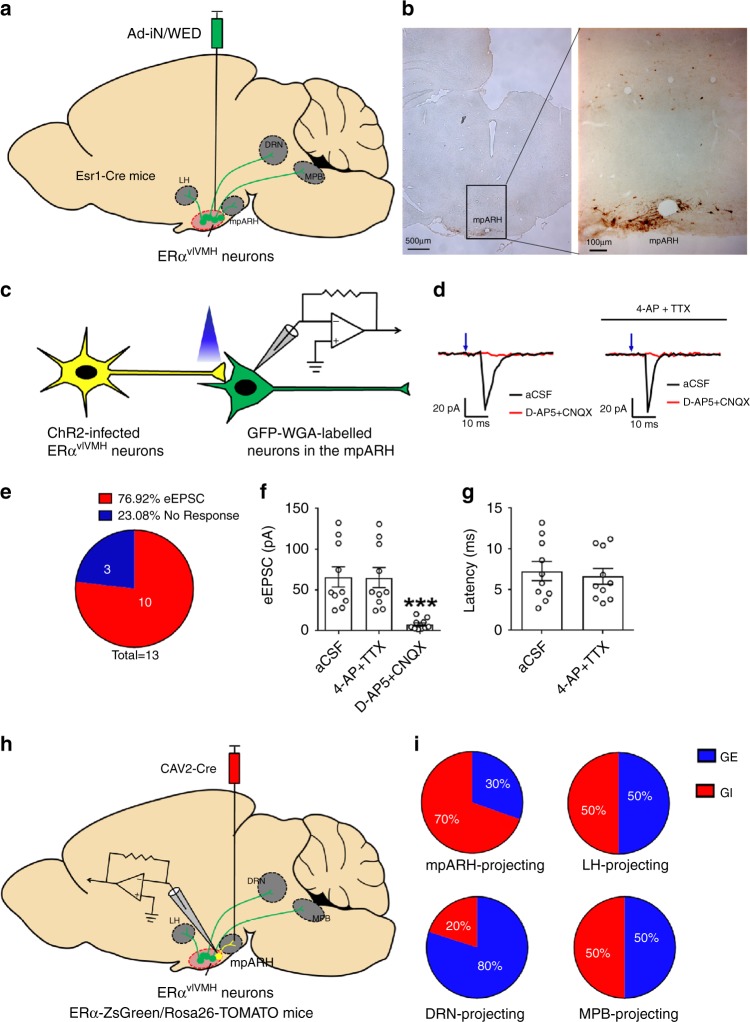
Projecting sites of GE- and GI-ERα^vlVMH^ neurons. **a** Schematic experimental strategy using the Ad-iN/WED virus as a transsynaptic anterograde tracer to identify downstream circuits of female ERα^vlVMH^ neurons. **b** Immunoreactivity of WGA in the mpARH. The right panel shows the higher magnification image of the black box in the left panel. Scale bars are indicated in each panel. The similar results were replicated three times. **c** Schematic experimental strategy for recordings in WGA-labeled neurons in response to photostimulation of ChR2-labeled ERα^vlVMH^-originated fibers within the mpARH in female mice. **d** Representative traces for light-evoked EPSCs, which were blocked by 30 μM D-AP5 and 50 μM CNQX, but not affected by 400 μM 4-AP and 1 μM TTX. **e** The percentage of WGA (+) neurons in the mpARH that showed light-evoked EPSCs or no response. **f** Amplitude of evoked EPSCs. **g** Latency of evoked EPSCs. *N* = 10 neurons from two mice per group. Results are shown as mean ± SEM. ***P = 0.0009 vs. other groups in one-way ANOVA followed by post hoc Sidak tests. **h** Schematic experimental strategy using the CAV2-Cre virus as a retrograde tracer to map GI-ERα^vlVMH^ and GE-ERα^vlVMH^-originated neural circuits. **i** Composition of GI and GE neurons in the female ERα^vlVMH^ populations with specific projections to the mpARH, the LH, the DRN, and the MPB. *N* = 14–23 neurons from three mice per projecting site. Source data are provided as a Source Data Fig. 4.

In order to examine the projection sites of ERα^vlVMH^ neurons, we stereotaxically injected Ad-iN/WED^22^ into the vlVMH of female Esr1-Cre mice. These mice expressed GFP-tagged wheat germ agglutinin (GFP-WGA, an anterograde transsynaptic tracer) specifically in ERα^vlVMH^ neurons (Fig. 4a). Abundant WGA-labeled neurons were detected in a few brain regions implicated in the regulation of energy and glucose homeostasis, including the medioposterior part of the arcuate hypothalamic nucleus (mpARH), the lateral hypothalamus (LH), the medial parabrachial nucleus (MPB), and the dorsal Raphe nuclei (DRN; Fig. 4b, Supplementary Fig. 3a, g, m). These results suggest that ERα^vlVMH^ neurons project to these distant brain regions.

Since WGA may also travel retrogradely^23^, the WGA-labeled neurons may not be the synaptic targets of ERα^vlVMH^ neurons. To further confirm the synaptic connectivity between ERα^vlVMH^ neurons and mpARH neurons, we stereotaxically injected Ad-iN/WED and AAV-EF1α-DIO hChR2(H134R)-EYFP into the vlVMH of female Esr1-Cre mice to express both channelrhodopsin-2 (ChR2)-EYFP and GFP-WGA specifically in ERα^vlVMH^ neurons. Angular brain slices (containing both the vlVMH and mpARH) were prepared from these mice to perform electrophysiological recordings in WGA-GFP-labeled mpARH neurons (Fig. 4c). We detected light-evoked excitatory postsynaptic currents (EPSCs) in 10 out of 13 WGA-GFP-labeled neurons (averaged 65.97 ± 12.52 pA, latency: 7.26 ± 1.16 ms; *n* = 10). These evoked EPSCs were blocked by NMDA and AMPA glutamate receptor antagonists, 30 µM D-AP5 and 30 µM CNQX (Fig. 4d, f), confirming the glutamatergic nature of these evoked EPSCs. Importantly, all evoked EPSC persisted in the presence of 400 µM 4-AP and 1 µM TTX (Fig. 4d, f, g), indicating that these neurotransmissions are likely monosynaptic. We performed similar electrophysiological recordings to demonstrate functional connectivity between ERα^vlVMH^ neurons and WGA-GFP-labeled neurons in the LH (Supplementary Fig. 3b–f), the DRN (Supplementary Fig. 3h–l), and the MPB (Supplementary Fig. 3n–r).

Then, to further determine whether these projections originate primarily from GI-ERα^vlVMH^ neurons or from GE-ERα^vlVMH^ neurons, we stereotaxically injected a retrograde CAV2-Cre virus^24^ into each of these four sites of female ERα-ZsGreen/Rosa26-TOMATO mice. In this case, CAV2-Cre retrogradely infected upstream neurons that project to the injection site (e.g., mpARH) and induced TOMATO (red fluorescence) expression in these mpARH-projecting neurons (Fig. 4h). Thus, in the vlVMH, those neurons double labeled by TOMATO and ZsGreen were identified as mpARH-projecting ERα^vlVMH^ neurons, which accounted for ~29.7% of ERα^vlVMH^ population (Supplementary Fig. 4a, b). Similarly, CAV2-Cre injected into the LH, DRN, or MPB induced TOMATO expression in ~22.6%, 15.0%, or 25.7%, respectively, of ERα^vlVMH^ neurons (Supplementary Fig. 4b). We further determined that ~71–84% TOMATO(+) neurons, retrogradely labeled by CAV2-Cre injected into one of the four respective regions, were ZsGreen(+) ERα^vlVMH^ neurons (Supplementary Fig. 4c). We then performed electrophysiology recordings in these TOMATO(+)/ZsGreen(+) neurons and determined whether they were GI or GE neurons (Fig. 4h). Interestingly, we found that the majority of mpARH-projecting ERα^vlVMH^ neurons are GI neurons, while the majority of DRN-projecting ERα^vlVMH^ neurons are GE neurons (Fig. 4i). On the other hand, ERα^vlVMH^ neurons that project to the LH and the MPB are mixtures of approximately equal numbers of GE and GI neurons (Fig. 4i).

**Fig. 5:**
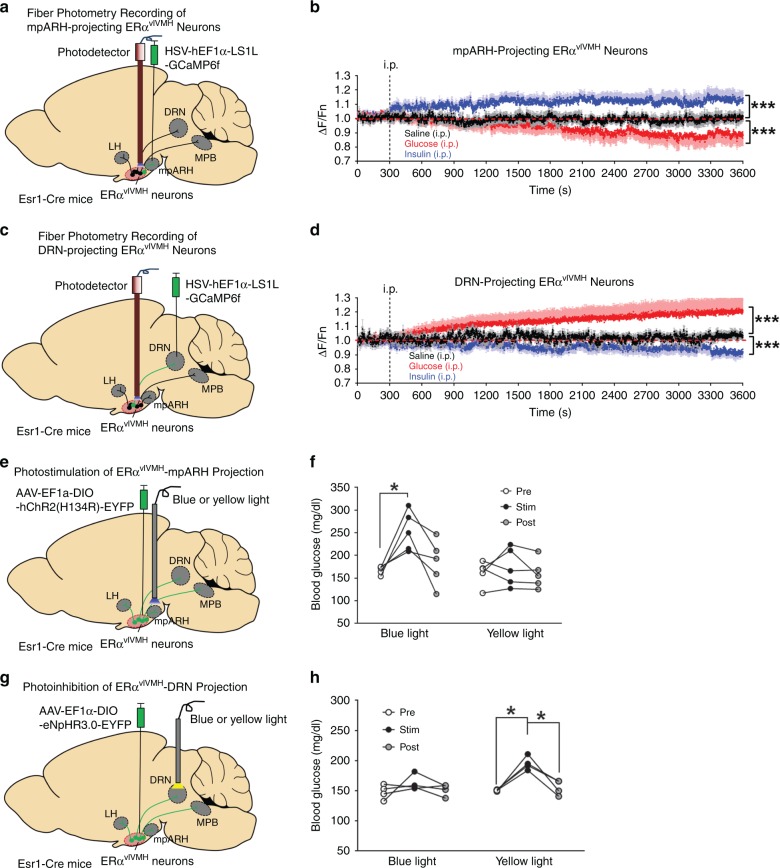
Distinct ERα^vlVMH^-downstream projections prevent hypoglycemia. a Schematic strategy using the HSV-hEF1α-LS1L-GCaMP6f virus and fiber photometry to monitor neural activity of female mpARH-projecting ERα^vlVMH^ neurons. **b** Quantifications of neural activity of mpARH-projecting ERα^vlVMH^ neurons in response to i.p. saline, i.p. 1 g per kg glucose, or i.p. 1.5 U per kg insulin. *N* = 6 mice per group. ***P < 0.0001 vs. saline group in two-way ANOVA analysis followed by post hoc Sidak tests. **c** Schematic strategy using the HSV-hEF1α-LS1L-GCaMP6f virus and fiber photometry to monitor neural activity of female DRN-projecting ERα^vlVMH^ neurons. **d** Quantifications of neural activity of DRN-projecting ERα^vlVMH^ neurons in response to i.p. saline, i.p. 1 g per kg glucose, or i.p. 1.5 U per kg insulin. *N* = 5 mice per group. ***P < 0.0001 vs. saline group in two-way ANOVA analysis followed by post hoc Sidak tests. **e** Schematic strategy using the AAV-EF1α-DIO hChR2(H134R)-EYFP to selectively activate the female ERα^vlVMH^→mpARH projection. **f** Effects of blue and yellow (as controls) light pulses on blood glucose. *N* = 5 mice per group. *P = 0.033 between the stimulation period and prestimulation period in one-way ANOVA analysis followed by post hoc Sidak tests. **g** Schematic strategy using the AAV-EF1α-DIO-eNpHR3.0-EYFP to selectively inhibit the female ERα^vlVMH^→DRN projection. **h** Effects of yellow and blue (as controls) light pulses on blood glucose. *N* = 4 mice per group. Between prestimulation period and stimulation period *P = 0.0111; between poststimulation period and stimulation period *P = 0.031 in one-way ANOVA analysis followed by post hoc Sidak tests. Results in **b** and **d** are shown as mean ± SEM. Source data are provided as a Source Data Fig. 5.

To further confirm these findings in vivo, we stereotaxically injected a retrograde virus HSV-hEF1α-LS1L-GCaMP6f into the mpARH of female Esr1-Cre mice and implanted a photodetector to target the vlVMH, which allowed fiber photometry recordings of the mpARH-projecting ERα^vlVMH^ neurons in functional mice (Fig. 5a). We showed that activity of mpARH-projecting ERα^vlVMH^ neurons was significantly reduced by hyperglycemia (i.p. 1 g per kg glucose) but increased by hypoglycemia (i.p. 1.5 U per kg insulin), confirming that these neurons were largely GI neurons (Fig. 5b). We used a similar approach to monitor activity of DRN-projecting ERα^vlVMH^ neurons, and found that these neurons displayed GE properties: activated by hyperglycemia but inhibited by hypoglycemia (Fig. 5c, d).

We further examined the glucose regulatory effects of mpARH-projecting ERα^vlVMH^ neurons. To this end, we stereotaxically injected AAV-EF1α-DIO hChR2(H134R)-EYFP into the vlVMH of female Esr1-Cre mice and implanted an optic fiber to target the mpARH (Fig. 5e, Supplementary Fig. 5a–c). Blue light pulses (473 nm, 5 ms per pulse, 40 pulses per 1 s for 1 h)^15^ were applied to this region to selectively activate the ERα^vlVMH^→mpARH projection (Supplementary Fig. 5d–f), which mimicked hypoglycemia-induced activation of this circuit. Interestingly, such activation resulted in significant increases in blood glucose (Fig. 5f). Importantly, as a control, yellow light pulses (589 nm, 5 ms per pulse, 40 pulses per 1 s for 1 h) applied to the same mice did not significantly alter blood glucose levels (Fig. 5f). On the other hand, the DRN-projecting ERα^vlVMH^ neurons are inhibited during a hypoglycemic event. Thus, we stereotaxically injected AAV-EF1α-DIO-eNpHR3.0-EYFP into the vlVMH of female Esr1-Cre mice and implanted an optic fiber to target the DRN (Fig. 5g, Supplementary Fig. 5g–j). Yellow light pulses were applied to the DRN to selectively inhibit the ERα^vlVMH^→DRN projection (Supplementary Fig. 5k–m), which also resulted in significant increases in blood glucose, while blue light pulses (as negative controls) showed no effect on glucose levels (Fig. 5h). Thus, these results support a feedback model that hypoglycemia activates GI-ERα^vlVMH^ neurons but inhibits GE-ERα^vlVMH^ neurons, which in turn engage distinct downstream neural circuits to increase blood glucose, and therefore prevent severe hypoglycemia.

Photostimulation of ERα^vlVMH^ neurons has been shown to evoke social investigation and occasional mounting in female mice when they are grouped housed^25^. In addition, activation of ERα^vlVMH^ neurons triggers aggressive behavior in Swiss Webster female mice, although this phenotype cannot be evoked in C57 females^26^. It is important to note that the current study measured glucose levels in singly housed mice, while examinations of social, sexual, and aggressive behaviors require animals to be encountered with an intruder or the opposite sex^25,26^. Nevertheless, we examined whether photostimulation of the ERα^vlVMH^→DRN circuit evokes similar social behaviors in singly housed female mice (all on the C57BL6/J background), but found no such behaviors (Supplementary Movie 1).

## Discussion

**Fig. 6:**
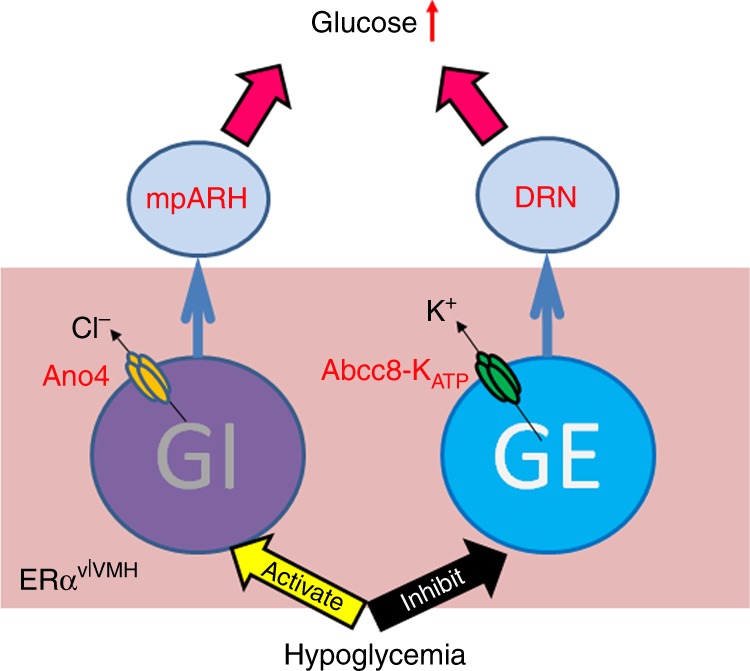
A schematic model. Hypoglycemia activates GI-ERα^vlVMH^ neurons through the opening of Ano4 channel, and inhibits GE-ERα^vlVMH^ neurons through the opening of the Abcc8-containing K_ATP_ channel. Activated GI-ERα^vlVMH^ neurons (via projections to the mpARH) and inhibited GE-ERα^vlVMH^ neurons (via projections to the DRN) both contribute to the prevention of severe hypoglycemia.

Our results identified ERα^vlVMH^ neurons as one key glucose-sensing neural population that can detect glucose fluctuations and prevent severe hypoglycemia at least in female mice. We further identified two key ion channels, namely the Ano4 channel and the K_ATP_ channel, which respectively mediate opposite GI and GE responses during the hypoglycemic challenge. Interestingly, subsets of GI- and GE-ERα^vlVMH^ neurons preferentially project to the mpARH and the DRN, respectively. Through these segregated downstream neural circuits, the opposite neural responses in these GI- and GE-ERα^vlVMH^ subsets are coordinated to synergistically elevate blood glucose, and therefore prevent severe hypoglycemia (Fig. 6).

Early work indicated that VMH neurons play essential roles in brain glucose sensing and the whole-body glucose balance^7–9,11^. Using genetic tools, recent research efforts started to reveal that distinct glucose-sensing subgroups within the VMH regulate glucose balance through diverse mechanisms. For example, the Friedman group showed that activation of glucokinase-expressing neurons in the dmVMH increases glucose^12^. Further, the Heisler group demonstrated that another subgroup in the dmVMH and cVMH indirectly sense glucose fluctuations, via neuronal inputs from glucose-sensing neurons in the PBN^14^. Consistently, the Morton group recently showed that dmVMH/cVMH neurons (marked by adult SF1 promoter) relay PBN synaptic inputs to defend against hypoglycemia^15^. It is important to note that while the entire VMH shares a common SF1 lineage, the vlVMH further differentiates into a neuronal cluster devoid of SF1 during the adulthood^27^. Thus, studies using adult SF1-Cre mice (e.g., receiving ChR2 virus injections) only target dmVMH and cVMH neurons, but not those in the vlVMH. Our studies used ERα as a molecular marker and demonstrated that ERα-expressing VMH neurons is a unique glucose-sensing population. Anatomically, these ERα neurons are highly concentrated within the vlVMH, but barely expressed in the dmVMH or cVMH. In addition, we showed that ERα^vlVMH^ neurons project to the mpARH, LH, MPB, and DRN. On the other hand, dmVMH/cVMH neurons (marked by the adult SF1 promoter) were reported to project to the anterior bed nucleus of stria terminlis, PVH, and central amygdala^15^. The uniqueness of ERα^vlVMH^ neurons also lies in their strong glucose-sensing capability. It has been previously reported that as a whole, ~50% of VMH neurons are capable of altering their firing activities in response to glucose fluctuations^28^. In line with these earlier findings, we found that 47–50% of dmVMH neurons and 46–48% of cVMH neurons are glucose sensing. Strikingly, we found that 100% of ERα^vlVMH^ neurons (in both male and female mice) that we tested are glucose sensing. Thus, ERα^vlVMH^ neurons represent a unique subpopulation with strong glucose-sensing capability.

It is intriguing that GI- and GE-ERα^vlVMH^ neurons displayed exactly opposite neural responses to hypoglycemia. In particular, GI neurons rapidly increase their activities in response to low glucose, despite the fact that glucose is a basic fuel for neuronal viability and functions. In searching for ionic mechanisms that regulate activities of GI-ERα^vlVMH^ neurons, we found that GI-ERα^vlVMH^ neurons, but not GE-ERα^vlVMH^ neurons, express high levels of Ano4 (a chloride channel) and display robust rectifying Ano currents. Interestingly, hypoglycemia can potentiate these currents that likely account for activation of GI-ERα^vlVMH^ neurons. Supporting this notion, both pharmacological blockade of Ano and CRISPR-mediated disruption of Ano4 abolish responses of GI-ERα^vlVMH^ neurons, but have no effect on GE-ERα^vlVMH^ neurons. Together, these results identified Ano4 as a key ion channel that mediates the activation of GI-ERα^vlVMH^ neurons evoked by hypoglycemia. Notably, NTS neurons marked by GLUT2, another GI population, respond to hypoglycemia through leak potassium conductances but not chloride conductances^5^, suggesting that diverse ionic mechanisms exist for GI populations in different brain regions.

On the other hand, the ion channel mediating neural responses in GE neurons appear to be conserved among various GE populations. In particular, the K_ATP_ channel has been reported to mediate glucose-sensing functions of GE neurons in the VMH, in the NTS and in the supraoptic nuclei^11,29–31^. Consistent with this notion, we found that GE-ERα^vlVMH^ neurons express high levels of Abcc8 (encoding the K_ATP_ channel subunit, Sur1) and display elevated K_ATP_ currents in response to hypoglycemia; blockade of K_ATP_ currents abolishes the hypoglycemia-induced inhibition in these neurons. Notably, GI-ERα^vlVMH^ neurons express minimal Abcc8, and hypoglycemia does not enhance K_ATP_ currents in these neurons. We further demonstrated the specific functions of Abcc8 in GE-ERα^vlVMH^ neurons. Thus, CRISPR-mediated disruption of Abcc8 reduces the K_ATP_ current and diminishes glucose-sensing capability of GE-ERα^vlVMH^ neurons, but does not affect GI-ERα^vlVMH^ neurons. Together, our results indicate that distinct ionic conductances mediate the opposite neural responses to glucose fluctuations in GI- vs. GE-ERα^vlVMH^ neurons. While our data indicate that the glucose-sensing activity of ERα^vlVMH^ neurons is important for preventing hypoglycemia, we cannot exclude the possibility that ERα^vlVMH^ neurons or other VMH neurons may relay glucose-sensing signals coming from outside of the VMH (or even outside of the brain) to regulate glucose balance.

Interestingly, subsets of GI- and GE-ERα^vlVMH^ neurons have different projection patterns. The mpARH-projecting ERα^vlVMH^ neurons are largely GI, whereas the DRN-projecting ERα^vlVMH^ neurons are largely GE in nature. This segregation was observed by both ex vivo slice electrophysiology and by in vivo fiber photometry. Then, we used the optogenetic approach to specifically activate the ERα^vlVMH^→mpARH projection or to inhibit the ERα^vlVMH^→DRN projection, which mimic their natural responses to hypoglycemia. Interestingly, these optogenetic manipulations both lead to significant increases in blood glucose. Thus, these results indicate that while the activities of GI-ERα^vlVMH^-originated and GE-ERα^vlVMH^-originated neural circuits are oppositely regulated by hypoglycemia, the functions of these neural circuits are coordinated to provide a synergistic response to restore the glucose balance.

Notably, in addition to the regulating metabolic balance^32–34^, ERα^vlVMH^ neurons have been implicated in multiple social behaviors, including investigation, mating, and aggression^25,26^. While we did not detect these social behaviors in singly housed mice with the ERα^vlVMH^→DRN circuit stimulated, our results cannot fully exclude the possibility that functions of ERα^vlVMH^ neurons on social behaviors may have influenced the overall outcome on the glucose homeostasis.

## Methods

### Mice

Several transgenic mouse lines, including ERα-ZsGreen, ERα-ZsGreen/Rosa26-TOMATO, and Esr1-Cre were maintained on a C57BL6/J background. Esr1-Cre mice were purchased from Jackson Laboratory (#017911) that express Cre recombinase selectively in ERα-expressing neurons, including those in the vlVMH^35^. In addition, some C57Bl6J mice were purchased from the mouse facility of Baylor College of Medicine. Mice were housed in a temperature-controlled environment at 22–24 ˚C using a 12 h light/12 h dark cycle. The mice were fed standard chow (6.5% fat, #2920, Harlan-Teklad, Madison, WI). Water was provided ad libitum. Further information about resources and reagents can be found in Table 1.

**Table 1:** Key resources table.

Reagent or resource	Source	Identifier
Antibodies		
Goat anti-WGA antibody	VectorLabs	AS-2024
Biotinylated donkey anti-goat secondary antibody	Jackson ImmunoResearch	705-065-003
Chemicals, peptides, and recombinant proteins		
Angiotensin II	Sigma	A9525
CaCCinh-A01	Tocris	4877
Tolbutamide	Sigma	T0891
TTX	Tocris	1078
4-AP	Tocris	0940
Insulin	Lily	HI-210
Glucose	Sigma	G8270
2-DG	Sigma	D6134
D-AP5	Tocris	0106
Bicuculline	Tocris	0131
Experimental models: organisms/strains		
Mouse: Esr1-Cre	Jackson Laboratory	017911
Mouse: ERα-ZsGreen	Saito, et al.^17^	N/A
Mouse: Rosa26-tdTOMATO	Jackson Laboratory	007909
Recombinant DNA		
CAV2-Cre	Montpellier vector platform	N/A
AAV8-FLEX-scCas9	Vector Biolabs	7122
Ad-iN/WED	Leinninger, et al.^22^	N/A
AAV8-EF1α-DIO hChR2(H134R)-EYFP	UNC Gene Therapy Center	AV4378G
AAV8-EF1α-DIO-eNpHR3.0-EYFP	UNC Gene Therapy Center	AV4846C
AAV8-Abcc8/sgRNAs-FLEX-tdTOMATO	This paper	N/A
AAV8-Ano4/sgRNAs-FLEX-tdTOMATO	This paper	N/A
HSV-hEF1α-LS1L-GCaMP6f	MGH GDT Core	RN506 (HT)

### Electrophysiology

ERα-ZsGreen or ERα-ZsGreen/Rosa26-TOMATO mice were used for electrophysiological recordings. Mice were deeply anesthetized with isoflurane and transcardially perfused with a modified ice-cold sucrose-based cutting solution (pH 7.3) containing 10 mM NaCl, 25 mM NaHCO_3_, 195 mM sucrose, 5 mM glucose, 2.5 mM KCl, 1.25 mM NaH2PO_4_, 2 mM Na-pyruvate, 0.5 mM CaCl_2_, and 7 mM MgCl_2_, bubbled continuously with 95% O_2_ and 5% CO_2_ (ref. ^36^). The mice were then decapitated, and the entire brain was removed and immediately submerged in the cutting solution. Slices (250 µm) were cut with a Microm HM 650 V vibratome (Thermo Scientific). Three brain slices containing the VMH were obtained for each animal (bregma −2.06 mm to −1.46 mm; interaural 1.74–2.34 mm). The slices were recovered for 1 h at 34 °C and then maintained at room temperature in artificial cerebrospinal fluid (aCSF, pH 7.3) containing 126 mM NaCl, 2.5 mM KCl, 2.4 mM CaCl_2_, 1.2 mM NaH2PO_4_, 1.2 mM MgCl_2_, 5.0 mM glucose, and 21.4 mM NaHCO_3_) saturated with 95% O_2_ and 5% CO_2_ before recording.

Slices were transferred to a recording chamber and allowed to equilibrate for at least 10 min before recording. The slices were superfused at 34 °C in oxygenated aCSF at a flow rate of 1.8–2 ml/min. ZsGreen and/or TOMATO-labeled neurons in the VMH were visualized using epifluorescence and IR-DIC imaging on an upright microscope (Eclipse FN-1, Nikon) equipped with a movable stage (MP-285, Sutter Instrument). Patch pipettes with resistances of 3–5 MΩ were filled with intracellular solution (pH 7.3) containing 128 mM K-gluconate, 10 mM KCl, 10 mM HEPES, 0.1 mM EGTA, 2 mM MgCl_2_, 0.05 mM Na-GTP, and 0.05 mM Mg-ATP. Recordings were made using a MultiClamp 700B amplifier (Axon Instrument), sampled using Digidata 1440 A and analyzed offline with pClamp 10.3 software (Axon Instruments). Series resistance was monitored during the recording, and the values were generally <10 MΩ and were not compensated. The liquid junction potential was +12.5 mV, and was corrected after the experiment. Data were excluded if the series resistance increased dramatically during the experiment or without overshoot for action potential. Currents were amplified, filtered at 1 kHz, and digitized at 20 kHz. Current clamp was engaged to test neural firing frequency and resting membrane potential at the baseline 5 mM glucose aCSF and 1 mM glucose aCSF. The values for resting membrane potential and firing frequency averaged within 2-min bin at the 5 mM glucose or 1 mM glucose aCSF condition. A neuron was considered depolarized or hyperpolarized if a change in membrane potential was at least 2 mV in amplitude.

To measure Ano currents, the pipette solution contained (in mM): CsCl 130, NaH_2_PO_4_ 1.2, Na_2_HPO_4_ 4.8, EGTA, MgCl_2_ 1.0, D-glucose 5.0, and ATP 3.0 (pH adjusted to 7.2). Total Ano current was recorded under voltage-clamp by holding the membrane potential at −60 mV in 5 mM glucose or 1 mM glucose aCSF. At intervals, neurons were voltage clamped from −50 mV to +50 mV in steps of 10 mV for 1 s (ref. ^37^). Then the neurons were treated with 100 μM CaCCinh-A01 (an Ano blocker) for 3 min (ref. ^38^). The Ano current was calculated by subtracting the left current in the presence of Ano blocker from total current without the blocker.

For the K_ATP_ current, slices were perfused with an external solution that contained 140 mM NaCl, 5 mM KCl, 2 mM CaCl_2_, 1 mM MgCl_2_, 5 mM glucose, and 10 mM HEPES (pH 7.4). The pipette (intracellular) solution contained 130 mM potassium gluconate, 20 mM HEPES, 10 mM EGTA, 1 mM MgCl_2_, 2.5 mM CaCl_2_, 1.0 mM Mg-ATP, and 0.3 mM Tris-GTP (pH 7.2)^39^. The neural membrane potential was hold at −60mV in voltage-clamp model when K_ATP_ current was recorded and when glucose was changed from 5 mM to 1 mM. K_ATP_ current was calculated by subtracting currents recorded in the absence and the presence of a K_ATP_ blocker, tolbutamide (200 µM)^18^.

### Patch-seq and data analysis

In order to examine gene expression profiling of GI-ERα^vlVMH^ or GE-ERα^vlVMH^ neurons, we first performed electrophysiological recordings to identify GI-ERα^vlVMH^ or GE-ERα^vlVMH^ neurons. Four GI-ERα^vlVMH^ neurons and four GE-ERα^vlVMH^ neurons (from two different mice) were manually collected and suspended in PBS buffer. Cell lysis, first-strand cDNA synthesis and cDNA amplification were performed according to the manufacturer’s instructions of SMART-Seq v4 Ultra Low Input RNA Kit (Clontech). Amplified cDNA was purified by Agencourt AMPure XP Kit (Beckman Coulter). The Nextera Library Prep Kit (Illumina) was used to prepare libraries for sequencing (100 bp paired-end, RNA-seq) on a HiSeq 2500 platform (Illumina).

RNA-seq raw data files were trimmed using TrimGalore (version 0.4.1) and aligned against the mouse reference genome assembly (GRCm38.p6) using the STAR aligner (version 2.5.3a)^40^. One sample (“GIneuron3”) was removed from the following analysis due to low sequence read counts (Supplementary Table 1). Next, gene expression was assessed using featureCounts (version 1.6.0)^41^. To improve the identification of differentially regulated genes, unwanted variation between samples was removed using RUVseq^42^. Then, DESeq2 was used to determine differential gene expression^43^. Significance of differential expression was assessed by requiring both *P* < 0.05 and |log_2_(fold change)| > 2. We found a total of 372 differentially expressed genes (Supplementary Data 1, Supplementary Fig. 2a). Gene set enrichment analysis was performed using the online tool WebGestalt (version 2019)^44^. Only the gene sets whose size was in a range of 5–2000 were considered and enrichment *P*-values were corrected for multiple testing by Benjamini–Hochberg procedure, as implemented in the tool.

### Real-time RT-qPCR analyses

To confirm the expression of Abcc8 and Ano4 in GE-ERα^vlVMH^ and GI-ERα^vlVMH^ neurons, respectively, we manually picked up identified GE or GI ZsGreen-labeled vlVMH neurons from female ERα-ZsGreen mice (at diestrus). To this end, the mouse brain was removed and immediately submerged in ice-cold sucrose-based cutting solution (adjusted to pH 7.3) containing (in mM) 10 NaCl, 25 NaHCO_3_, 195 sucrose, 5 glucose, 2.5 KCl, 1.25 NaH_2_PO_4_, 2 Na-pyruvate, 0.5 CaCl_2_, and 7 MgCl_2_ bubbled continuously with 95% O_2_ and 5% CO_2_. The slices (250 μm) were cut with a Microm HM 650 V vibratome (Thermo Scientific) and recovered for 1 h at 34 °C, and then maintained at room temperature in artificial cerebrospinal fluid (aCSF, pH 7.3) containing 126 mM NaCl, 2.5 mM KCl, 2.4 mM CaCl_2_, 1.2 mM NaH_2_PO_4_, 1.2 mM MgCl_2_, 11.1 mM glucose, and 21.4 mM NaHCO_3_ saturated with 95% O_2_ and 5% CO_2_ before recording. Slices were transferred to a chamber, and ZsGreen-labeled neurons were visualized using epifluorescence and IR-DIC imaging on an upright microscope equipped with a moveable stage (MP-285, Sutter Instrument). These neurons were first subjected to electrophysiological recordings to determine whether they were GE neurons or GI neurons, as described above. Single neurons were then manually picked up by the pipette and subjected to RNA extraction and reverse transcription using the Ambion Single-Cell-to-CT Kit (Ambion, Life Technologies) according to the manufacturer’s instruction. Briefly, 10 μl Single Cell Lysis solutions with DNase I was added to each sample, and the supernatant after centrifuge were used for cDNA synthesis (25 °C for 10 min, 42 °C for 60 min, and 85 °C for 5 min). The cDNA samples were amplified on a CFX384 Real-Time System (Bio-Rad) using SsoADV SYBR Green Supermix (Bio-Rad), and data were collected using Bio-Rad CFX Manager (3.1). Results were normalized against the expression of house-keeping gene (cyclophilin). Primer sequences were listed in Supplementary Table 3.

### CRISRP-Cas9 deletion of Ano4 and Abcc8

AAV vectors carrying sgRNAs targeting mouse Ano4 or Abcc8 were designed and constructed by Biocytogen (Wakefield, MA). For Ano4, exon 4 and exon 11 were chosen to be targeted by CRISPR-Cas9. A total of 19 sgRNAs were designed with seven targeting exon 4 and 12 targeting exon 11. These sgRNAs were selected using the CRISPR tool (https://www.sanger.ac.uk/htgt/wge/) with minimal potential off-target effects. All 19 sgRNAs were screened for on-target activity using a Universal CRISPR Activity Assay (UCA^TM^, Biocytogen)^45^. Briefly, the plasmid carrying Cas9 and sgRNA, and another plasmid carrying the target sequence cloned inside a luciferase gene were co-transfected into HEK293. Stop codon and CRISPR/Cas9 targeting sites were located within the luciferase gene. Stop codon induced the translational termination of the luciferase gene, while sgRNA targeting site cutting induced DNA annealing based on single-strand annealing and the complementary sequence recombination thereby occurred to rescue a complete coding sequence of the luciferase. The luciferase signal was then detected to reflect the DNA editing efficiency of the sgRNA. We used the pCS(puro)-positive plasmid, which expressed a proven positive-sgRNA, as the positive control (Supplementary Fig. 2b). The sgRNA#2 (GCACTTCGGAGGACACCAGC **AGG)** and sgRNA#9-A (GTACTTGTACCACACGCCCC **AGG**) were selected to target exon 4 and exon 11 of the Ano4 gene, respectively, due to their relatively high on-target activity and low off-target potentials. Similarly for Abcc8, exon 2 and exon 5 were chosen to be targeted by CRISPR-Cas9. A total of 14 sgRNAs were designed with seven targeting exon 2 and 7 targeting exon 5. These sgRNAs were selected using the CRISPR tool (https://www.sanger.ac.uk/htgt/wge/) with minimal potential off-target effects. All 14 sgRNAs were screened for on-target activity using the UCA^TM^ Assay (Supplementary Fig. 2d). The sgRNA#5 (TGAAGGTAAGGATCCAGCGC **AGG)** and sgRNA#11 (GCAGCTTCCCGATGGCCCGC **AGG**) were used for next step. We constructed an AAV-U6-sgRNA-tdTomato vector containing two sgRNAs targeting Ano4 (AAV-Ano4/sgRNAs-FLEX-tdTOMATO) or two sgRNAs targeting Abcc8 gene (AAV-Abcc8/sgRNAs-FLEX-tdTOMATO), respectively (Supplementary Fig. 2c, e). Briefly, U6 promoter-sgRNAs, CAG promoter-flex-tdTomato-bGH polyA cassettes were cloned into the Addgene plasmid #61591 vector (http://www.addgene.org/61591/) and further verified by full sequencing. The two viruses were packaged by the Baylor IDDRC Neuroconnectivity Core.

To validate AAV-Ano4/sgRNAs-FLEX-tdTOMATO in mice, female Esr1-Cre mice (12 weeks of age) received stereotaxic injections of AAV-FLEX-scCas9 (80 nl, 5.3 × 10^12^ GC per ml) and AAV-Ano4/sgRNAs-FLEX-tdTOMATO (160 nl, 1.4 × 10^12^ GC per ml) into one side of the vlVMH (knockout side), and received AAV-Ano4/sgRNAs-FLEX-tdTOMATO (160 nl) and the AAV-GFP (80 nl, 5.6 × 10^12^ GC per ml, no Cas9) in the other side of the vlVMH virus (control side). After a 4-week recovery, these mice were subjected to electrophysiology recordings for glucose-sensing properties and Ano currents, as described above. Since we did not find any GI neurons from the knockout side, we measured Ano currents in non-GE neurons from the knockout side (which were likely original GI neurons) and compared these currents to Ano currents in identified GI neurons from the control side.

Similarly, to validate AAV-Abcc8/sgRNAs-FLEX-tdTOMATO in mice, female Esr1-Cre mice received stereotaxic injections of AAV-FLEX-scCas9 (80 nl) and AAV-Abcc8/sgRNAs-FLEX-tdTOMATO (160 nl, 1.3 × 10^12^ GC per ml) into one side of the vlVMH (knockout side), and received AAV-Abcc8/sgRNAs-FLEX-tdTOMATO (160 nl) and the AAV-GFP (80 nl, no Cas9) in the other side of the vlVMH virus (control side). After a 4-week recovery, these mice were subjected to electrophysiology recordings for glucose-sensing properties and K_ATP_ currents, as described above. Since we did not find any GE neurons from the knockout side, we measured K_ATP_ currents in non-GI neurons from the knockout side (which were likely original GE neurons) and compared these currents to K_ATP_ currents in identified GE neurons from the control side.

### Icv 2-DG assay

To determine the functions of Ano4 and Abcc8 in ERα^vlVMH^ neurons in vivo, female wild type and Esr1-Cre littermates (12 weeks of age) received stereotaxic injections of AAV-FLEX-scCas9 and AAV-Ano4/sgRNAs-FLEX-tdTOMATO into both sides of the vlVMH (1.60 mm posterior, 0.70 mm lateral, and 5.90 mm ventral to the bregma, based on Franklin and Paxinos Mouse Brain Atlas). During the same surgeries, an indwelling icv guide cannula (#62003, Plastics One) was stereotaxically inserted to target the lateral ventricle (0.34 mm posterior, 1.00 mm lateral, and 2.30 mm ventral to the bregma). One week after surgery, the cannulation accuracy was validated by central administration of 10 ng angiotensin II (A9525, Sigma), which induced the increase of drinking and grooming behavior. Mice were subjected to weekly handling to adapt to stress associated with icv injections. Four weeks after the surgeries, mice were fasted for 3 h from 9 a.m. in the morning. At 12 p.m., mice received icv injections of saline or 2-DG (1 mg in 2 μl saline). Blood glucose was then measured at 0, 30, 60, and 120 min after injections.

### WGA anterograde tracing

In order to map the downstream neurons of ERα^vlVMH^ neurons, 12-week-old Esr1-Cre female mice were anesthetized by isoflurane and received stereotaxic injections of Ad-FLEX-WGA-EGFP^22^ into the vlVMH (200 nl, 6.1 × 10^12^ VP per ml; 1.60 mm posterior, 0.70 mm lateral, and 5.90 mm ventral to the bregma, based on Franklin and Paxinos Mouse Brain Atlas). Because Ad-iN/WED is Cre-dependent virus, WGA-GFP was exclusively expressed in ERα^vlVMH^ neurons and anterogradely traveled along the fibers, passed the synapse, and filled the downstream neurons that were innervated by ERα^vlVMH^ terminals. Four weeks after injections, mice were perfused with 10% formalin, and brain sections were cut at 25 μm (5 series). The sections were incubated at room temperature in primary goat anti-WGA antibody (1:1000, #AS-2024, VectorLabs) overnight, followed by biotinylated donkey anti-goat secondary antibody (1:1000; #705-065-003, Jackson ImmunoResearch) for 2 h. Sections were then incubated in the avidin–biotin complex (1:500, ABC; Vector Elite Kit), and incubated in 0.04% 3, 3′-diaminobenzidine and 0.01% hydrogen peroxide. After dehydration through graded ethanol, the slides were then immersed in xylene and coverslipped. Images were analyzed using a brightfield Leica microscope equipped with the Leica MM AF Acquisition and Analysis (#11640901).

### ChR2-assisted circuit mapping

We performed the WGA-ChR2-assisted circuit mapping^46–48^ in order to demonstrate the connectivity of ERα^vlVMH^ neurons and their downstream target neurons in various brain regions. Briefly, 12-week-old Esr1-Cre female mice were anesthetized by isoflurane and received stereotaxic injections of Ad-iN/WED (200 nl, 6.1 × 10^12^ VP per ml) and AAV-EF1α-DIO hChR2(H134R)-EYFP (200 nl, 6.2 × 10^12^ VP per ml) into the vlVMH. After a 4-week recovery, mice were sacrificed and brain slices (containing both the vlVMH and one of its target regions, e.g., LH, mpARH, DRN, or MPB) were prepared from these mice to perform electrophysiological recordings in WGA-GFP-labeled neurons in the target region. Neurons were patched using electrodes with tip resistances at 3.0–5.0 MΩ. Recording pipettes were routinely filled with a solution containing the following (in mM): 125 K-gluconate, 15 KCl, 10 HEPES, 8 NaCl, 4 Mg-ATP, 0.3 Na-GTP, 10 Na_2_-phosphocreatine, 2 EGTA, pH 7.30. The holding potential for voltage-clamp recordings was −60 mV, and responses were digitized at 10 kHz. All experiments were performed in the presence of GABA_A_ receptor antagonist bicuculline (50 μM). EPSCs were evoked by a 473 nm laser (C.N.I) to stimulate ChR2-expressing fibers every 20 s. D-AP5 (30 μM; an NMDA receptor antagonist) and CNQX (30 μM; an AMPA receptor antagonist) were added to confirm whether the light-evoked currents were glutamatergic synaptic currents. TTX (1 μm) and 4-AP (400 μM) were added to the aCSF in order to determine whether the response was monosynaptic.

### CAV2-Cre retrograde tracing and electrophysiology

In order to identify glucose-sensing neurons that send projections from the vlVMH to downstream brain regions, 12-week-old ERα-ZsGreen/Rosa26-TOMATO female mice were anesthetized by isoflurane and received unilateral stereotaxic injections of CAV2-Cre (200 nl, 3.7 × 10^12^ VP per ml) into one of the following sites: LH (1.06 mm posterior, 1.20 mm lateral, and 5.00 mm ventral to the bregma), mpARH (2.70 mm posterior, 0.25 mm lateral, and 5.60 mm ventral to the bregma), MPB (5.20 mm posterior, 1.25 mm lateral, and 3.80 mm ventral to the bregma), or DRN (4.65 mm posterior, 0 mm lateral, and 3.60 mm ventral to the bregma). CAV2 virus retrogradely traveled from the initial site to the brain region that project to the LH, mpARH, MPB, or DRN, and Cre recombinase induced TOMATO expression in these infected cells. Two weeks later, unfixed brain slices containing the VMH (150 µm in thickness) were prepared from these mice, and were subjected to fluorescent microscopy to visualize and quantify ZsGreen(+), TOMATO(+) and ZsGreen(+)/TOMATO(+) neurons in the vlVMH. About 500–600 neurons were counted from each mouse and two mice were included for each injection site (LH, mpARH, DRN, or MPB).

In parallel, some of CAV2-Cre-injected mice were used for electrophysiology. Briefly, whole-cell patch-clamp recordings were performed on identified dual fluorescent neurons (ZsGreen and TOMATO) in the brain slices containing the VMH. Current clamp was engaged to test neural firing frequency and resting membrane potential at the 5 mM glucose aCSF and 1 mM glucose aCSF, as described above, in order to identify them as GE or GI neurons. The composition of GE and GI neurons for each projecting site was calculated.

### Fiber photometry

For the fiber photometry experiments, Esr1-Cre female mice (12 weeks of age) were anesthetized by isoflurane and received stereotaxic injections of HSV-hEF1α-LS1L-GCaMP6f (200 nl per site, 3 × 10^9^ VP per ml) into the DRN or into the mpARH. During the same surgery, an optical fiber (fiber: core = 400 μm; 0.48 NA; M3 thread titanium receptacle; Doric Lenses) was implanted over the vlVMH (1.60 mm posterior, 0.70 mm lateral, and 5.70 mm ventral to the bregma, based on Franklin and Paxinos Mouse Brain Atlas). Fibers were fixed to the skull using dental acrylic and mice were allowed 3 weeks for recovery before acclimatization investigator handling for 1 week before experiments.

The fiber photometry recordings started 4–6 weeks after surgeries to allow for adequate recovery and GCaMP6f expression to stabilize. All recordings were done in the home cage of the singly housed experimental animal. Mice were allowed to adapt to the tethered patchcord for 2 days prior to experiments and given 5 min to acclimate to the tethered patchcord prior to any recording. Continuous <20 μW blue LED at 465 nm and UV LED at 405 nm served as excitation light sources, driven by a multichannel hub (Doric Lenses), modulated at 211 Hz and 330 Hz, respectively. The light was delivered to a filtered minicube (FMC5, Doric Lenses) before connecting through optic fibers to a rotary joint (FRJ 1 × 1, Doric Lenses) to allow for movement. GCaMP6f calcium GFP signals and UV autofluorescent signals were collected through the same fibers back to the dichroic ports of the minicube into a femtowatt silicon photoreceiver (2151, Newport). The digital signals were then amplified, demodulated, and collected through a lock-in amplifier (RZ5P, Tucker-Davis Technologies)^49^. The fiber photometry data was collected using Synapse 2.0 (Tucker-Davis Technologies) and down sampled to 8 Hz. We derived the values of fluorescence change (Δ*F*/*F*0) by calculating (*F*_465_ − *F*_405_)/*F*_465_ (ref. ^50^).

### Optogenetic in vivo studies

Esr1-Cre female mice (12 weeks of age) were anesthetized with isoflurane and received stereotaxic injections of Cre-dependent AAV expressing ChR2-YFP (AAV-EF1α-DIO hChR2(H134R)-YFP, 6.2 × 10^12^ VP per ml) or eNpHR3.0-EYFP (AAV-EF1α-DIO-eNpHR3.0-EYFP, 3 × 10^12^ VP per ml) into the vlVMH (200 nl; 1.60 mm posterior, 0.70 mm lateral, and 5.90 mm ventral to the bregma). Simultaneously, an optic fiber (0.2 mm in diameter with a numerical aperture of 0.22) was implanted to target mpARH (2.70 mm posterior, 0.25 mm lateral, and 5.45 mm ventral to the bregma) for ChR2-YFP-injected mice or the DRN (4.65 mm posterior, 0.00 mm lateral, and 3.25 mm ventral to the bregma) for eNpHR3.0-EYFP-injected mice. Importantly, the mpARH is very caudal (posterior) to the typical ARH where most of POMC and AgRP neurons are located. As shown in Fig. 4b, in the mpARH-containing coronal section that have WGA-labeled neurons, the third ventricle already becomes a small hole, namely mammillary recess of the third ventricle. According the mouse brain atlas, the mpARH is ~1.2 mm posterior to the vlVMH.

After a 7-day recovery, mice were fasted for 3 h from 9 a.m. in the morning to ensure empty stomach. At 12 p.m., blue or yellow light stimulation (473 nm or 589 nm, 5 ms per pulse, 40 pulses per 1 s for 1 h) was used to activate ERα^vlVMH^→mpARH or inhibit ERα^vlVMH^→DRN neural circuits as described by others^15^. Briefly, light intensity was applied at 21 mW per mm2 for photostimulation or 10 mW per mm^2^ for photoinhibition to reach appropriate light power exiting the fiber tip in the brain corresponding to 8 mW and 4 mW for activation and inhibition, respectively (web.stanford.eduper group/dlab/cgi-bin/graph/chart.php). Blood glucose was measured at three time points: prior to the start of photostimulation/inhibition, at the end of 1-h photostimulation/inhibition, and 1 h afterward.

To validate accurate and sufficient infection of ChR2-YFP or eNpHR3.0-EYFP in ERα^vlVMH^ neurons, all mice were perfused with 10% formalin. Brain sections were cut at 25 μm (5 series) and subjected to histological validation. Only those mice with YFP in the vlVMH, and the fiber tract in the mpARH or DRN were included in analyses.

### Statistical analyses

For electrophysiology recordings, the investigator was not blinded for animals’ genotypes, but he was blinded with the treatments (e.g., virus injections) the animals were subjected to. For the measurement of glucose, investigators were blinded for animal’s genotypes or the surgeries the animals were subjected to. For Patch-seq study, the investigator was blinded with the nature of neurons (GE or GI).

The data are presented as mean ± SEM (standard error of the mean). Statistical analyses were performed using GraphPad Prism 7.0 to evaluate normal distribution and variations within and among groups. Methods of statistical analyses were chosen based on the design of each experiment and are indicated in figure legends or main text. *P* < 0.05 was considered to be statistically significant.

### Study approval

Care of all animals and procedures were approved by the Baylor College of Medicine Institutional Animal Care and Use Committee.

### Reporting summary

Further information on research design is available in the Nature Research Reporting Summary linked to this article.

## Supplementary information

Supplementary Information

Description of Additional Supplementary Items

Supplementary Data 1

Supplementary Data 2

Supplementary Movie 1

Reporting summary

## Data Availability

The datasets generated during and/or analyzed during the current study are available from the corresponding author on reasonable request. The Patch-seq data were deposited in NCBI with a GEO ID of GSE146543.

## Source data

Source Data
