# Supplementary material for: Estrogen receptor-α expressing neurons in the ventrolateral VMH regulate glucose balance
Source: Nat Commun. 2020 May 1;11:2165. doi: 10.1038/s41467-020-15982-7 (PMC7195451; doi:10.1038/s41467-020-15982-7)
Supplement: Supplementary file 1 — Supplementary Information [file 41467_2020_15982_MOESM1_ESM.pdf]

Estrogen receptor-α expressing neurons in the ventrolateral VMH regulate glucose balance

He et al.

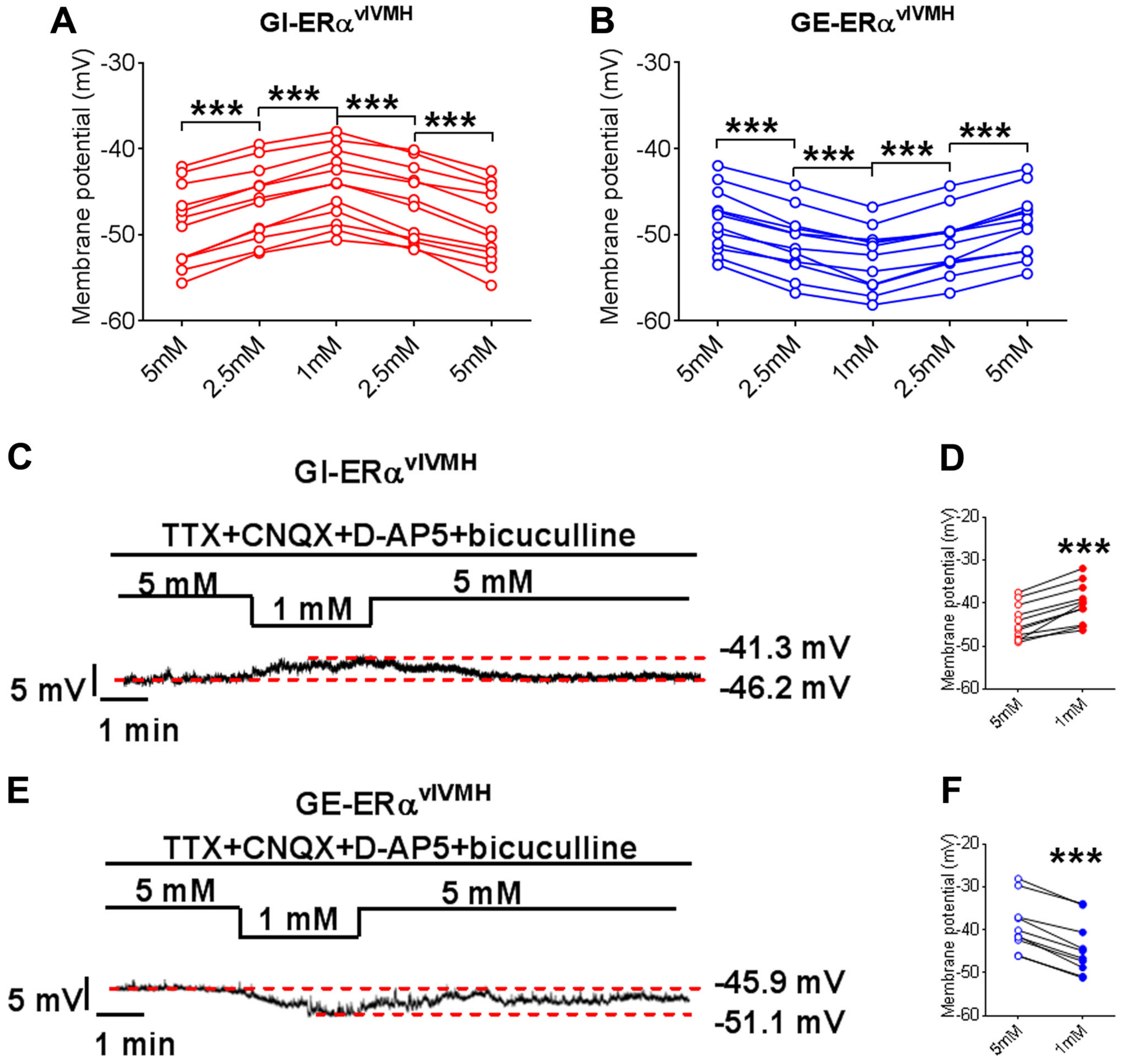

Supplementary Figure 1. Glucose-sensing of ERα^{vlVMH} neurons is independent of synaptic inputs (related to Figure 1). (A-B) Membrane potential of female GI-ERα^{vlVMH} neurons (A) and GE-ERα^{vlVMH} neurons (B) in response to 5→2.5→1→2.5→5 mM glucose fluctuations. N= 12 neurons from 3 mice/group. *** P<0.0001 in one way ANOVA followed by post hoc Sidak tests. (C and E) Representative electrophysiological responses to glucose fluctuations (5→1→5 mM) in the presence of 1 μM TTX, 30 μM CNQX, 30 μM D-AP5 and 50 μM bicuculline in a female GI-ERα^{vlVMH} neuron (C) or a female GE-ERα^{vlVMH} neuron (E). (D and F) Membrane potential of female GI-ERα^{vlVMH} neurons (D) and GE-ERα^{vlVMH} neurons (F) at 5 mM or 1 mM glucose condition in the presence of 1 μM TTX, 30 μM CNQX, 30 μM D-AP5 and 50 μM bicuculline. N= 10 or 11 neurons from 3 mice/group. *** P<0.0001 in two-sided paired t-tests. Source data are provided as a Source Data Supplementary Figure 1.

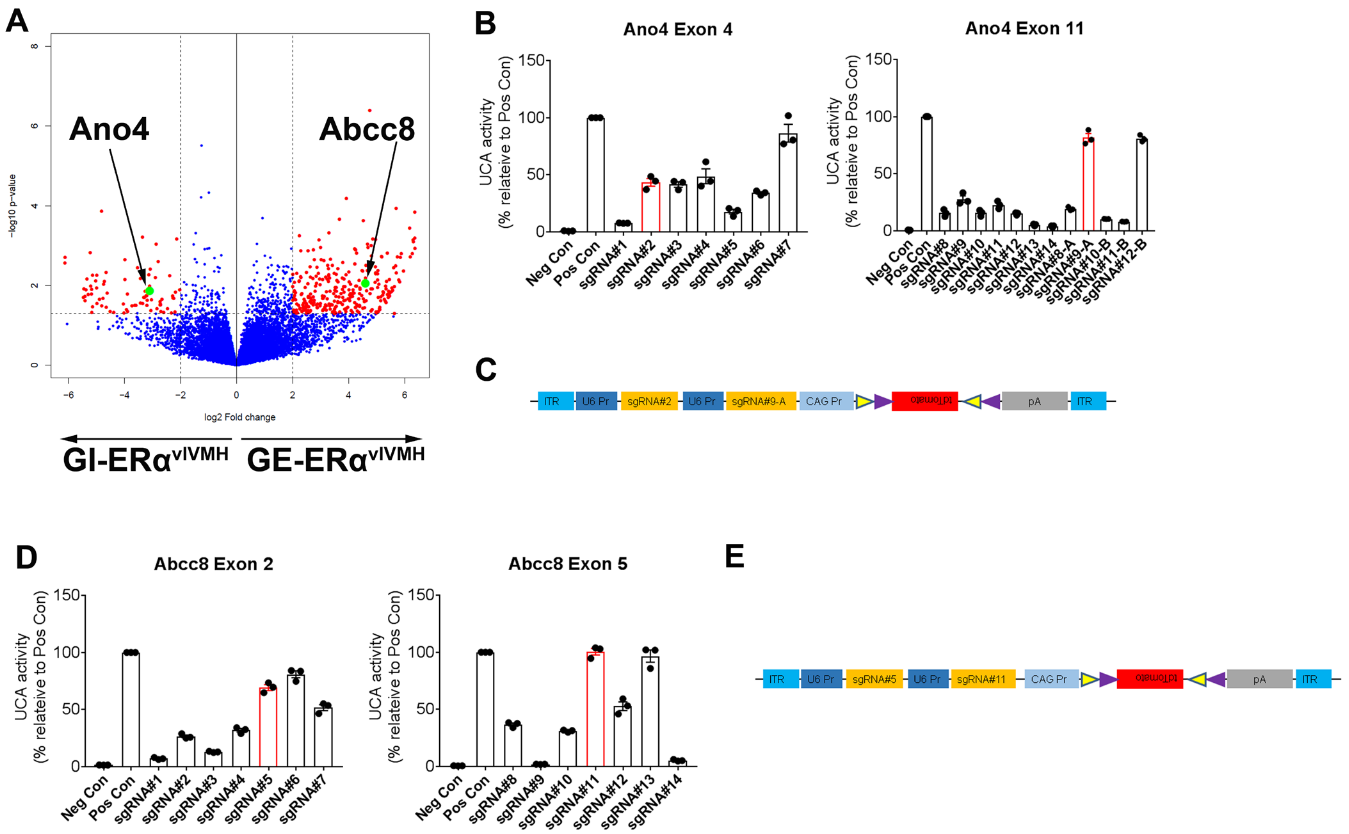

Supplementary Figure 2. Ano4 and Abcc8. (related to Figures 2-3). (A) Volcano plot showing differential gene expression by -$\log_{10}$(p value) and $\log_2$(fold change). Ano4 and Abcc8 are highlighted as GI- or GE-enriched gene, respectively. (B) Universal CRISPR Activity Assay (UCA) detecting DNA editing efficiency of 19 sgRNAs targeting exon 4 or 11 of Ano4 gene, respectively. Red-highlighted sgRNAs were selected to be included in the AAV construct. Note that sgRNA#2, with modest DNA editing efficiency, was selected due to its low off-target potential compared to other sgRNAs. N=3 independent experiments. (C) Schematic construction of the AAV vector carrying sgRNA#2, sgRNA#9-A and Cre-dependent tdTOMATO reporter. (D) Universal CRISPR Activity Assay (UCA) detecting DNA editing efficiency of 14 sgRNAs targeting exon 2 or 5 of Abcc8 gene, respectively. Red-highlighted sgRNAs were selected to be included in the AAV construct. N=3 independent experiments. (E) Schematic construction of the AAV vectors carrying sgRNA#5, sgRNA#11 and Cre-dependent tdTOMATO reporter. Results in (B and D) are shown as mean ± SEM. Source data are provided as a Source Data Supplementary Figure 2.

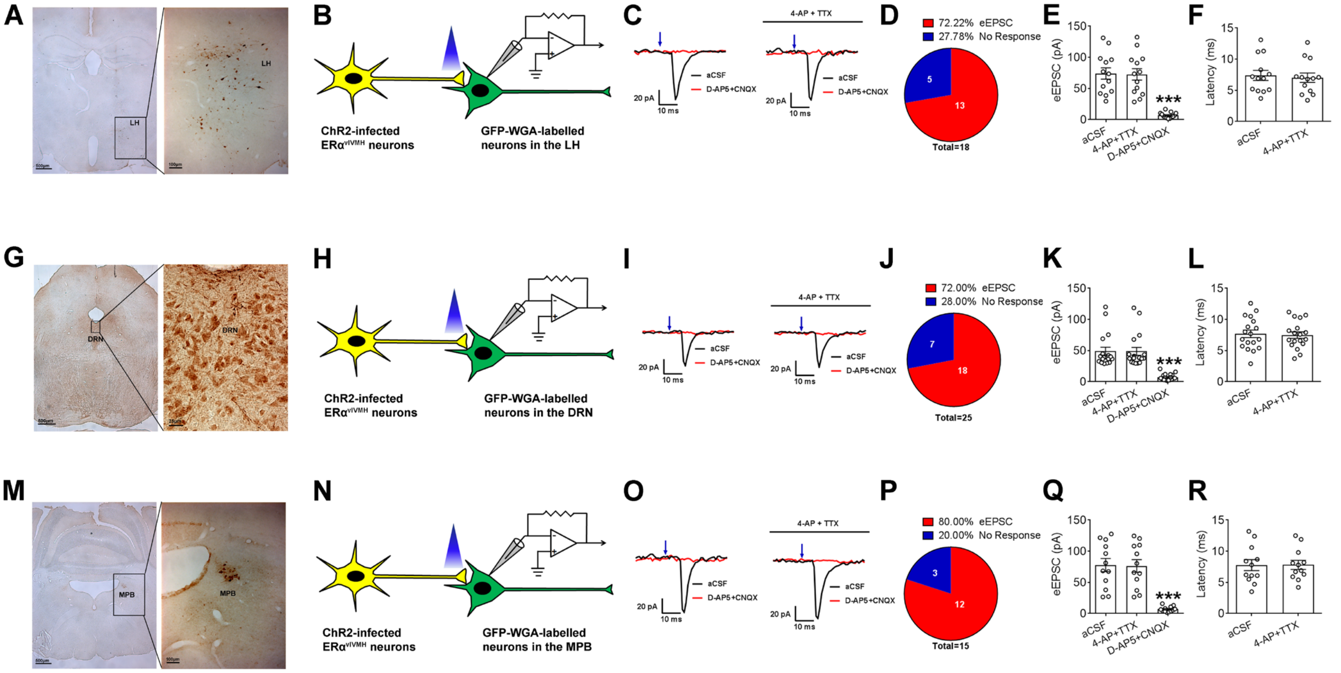

Supplementary Figure 3. Functional connectivity between ERα^{vlVMH} neurons and their targets (related to Figure 4). (A, G and M) Immunoreactivity of WGA in the LH (A), DRN (G) and MPB (M). The right panels show higher magnification images of the black box in the left panels. Scale bars are indicated in each panel. The similar results were replicated 3 times. (B, H, and N) Schematic experimental strategy for recordings in WGA-labelled neurons in response to photostimulation of ChR2-labelled ERα^{vlVMH}-originated fibers within the LH (B), DRN (H) and MPB (M) in female mice. (C) Representative traces for light-evoked EPSCs, which were blocked by 30 µM D-AP5 and 50 µM CNQX, but not affected by 400 µM 4-AP and 1 µM TTX. (D) The percentage of WGA (+) neurons in the LH that showed light-evoked EPSCs or no response. (E) Amplitude of evoked EPSCs. (F) Latency of evoked EPSCs. N=13 neurons from 2 mice per group. *** P<0.0001 vs. other groups in one-way ANOVA followed by post hoc Sidak tests. (I) Representative traces for light-evoked EPSCs, which were blocked by 30 µM D-AP5 and 50 µM CNQX, but not affected by 400 µM 4-AP and 1 µM TTX. (J) The percentage of WGA (+) neurons in the DRN that showed light-evoked EPSCs or no response. (K) Amplitude of evoked EPSCs. (L) Latency of evoked EPSCs. N=18 neurons from 3 mice per group. *** P<0.0001 vs. other groups in one-way ANOVA followed by post hoc Sidak tests. (O) Representative traces for light-evoked EPSCs, which were blocked by 30 µM D-AP5 and 50 µM CNQX, but not affected by 400 µM 4-AP and 1 µM TTX. (P) The percentage of WGA (+) neurons in the MPB that showed light-evoked EPSCs or no response. (Q) Amplitude of evoked EPSCs. (R) Latency of evoked EPSCs. N=12 neurons from 2 mice per group. *** P<0.0001 vs. other groups in one-way ANOVA followed by post hoc Sidak tests. Results in (E, F, K, L, Q, and R) are shown as mean ± SEM. Source data are provided as a Source Data Supplementary Figure 3.

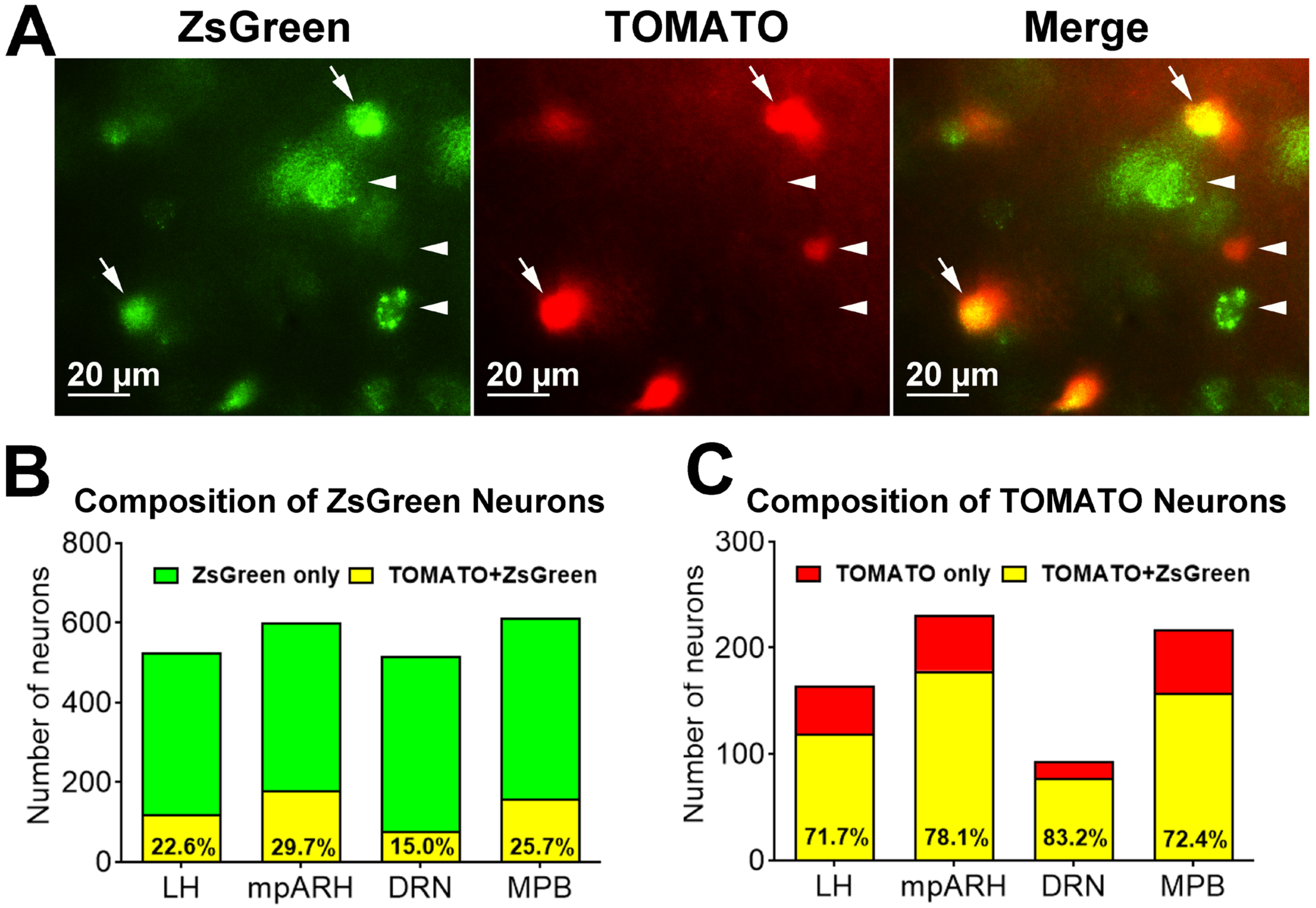

Supplementary Figure 4. Retrograde tracing from ERα^{vlVMH} target regions to the vlVMH (related to Figure 4). (A) Representative microscopic images showing ZsGreen(+), TOMATO(+) and ZsGreen(+)/TOMATO(+) neurons in the vlVMH of a brain slice from a female ERα-ZsGreen/Rosa26-TOMATO mouse receiving CAV2-Cre injected into the MPB. Arrows indicate double positive neurons; arrowheads indicate single positive neurons. (B) Composition of ZsGreen neurons (ZsGreen only or TOMATO/ZsGreen double positive) in female ERα-ZsGreen/Rosa26-TOMATO mice receiving CAV2-Cre injected into the LH, mpARH, DRN or MPB. Percentages of double positive neurons within the ZsGreen population were indicated. (C) Composition of TOMATO neurons (TOMATO only and TOMATO/ZsGreen double positive). Percentages of double positive neurons within the TOMATO population were indicated. Two mice were included for each injection site. Source data are provided as a Source Data Supplementary Figure 4.

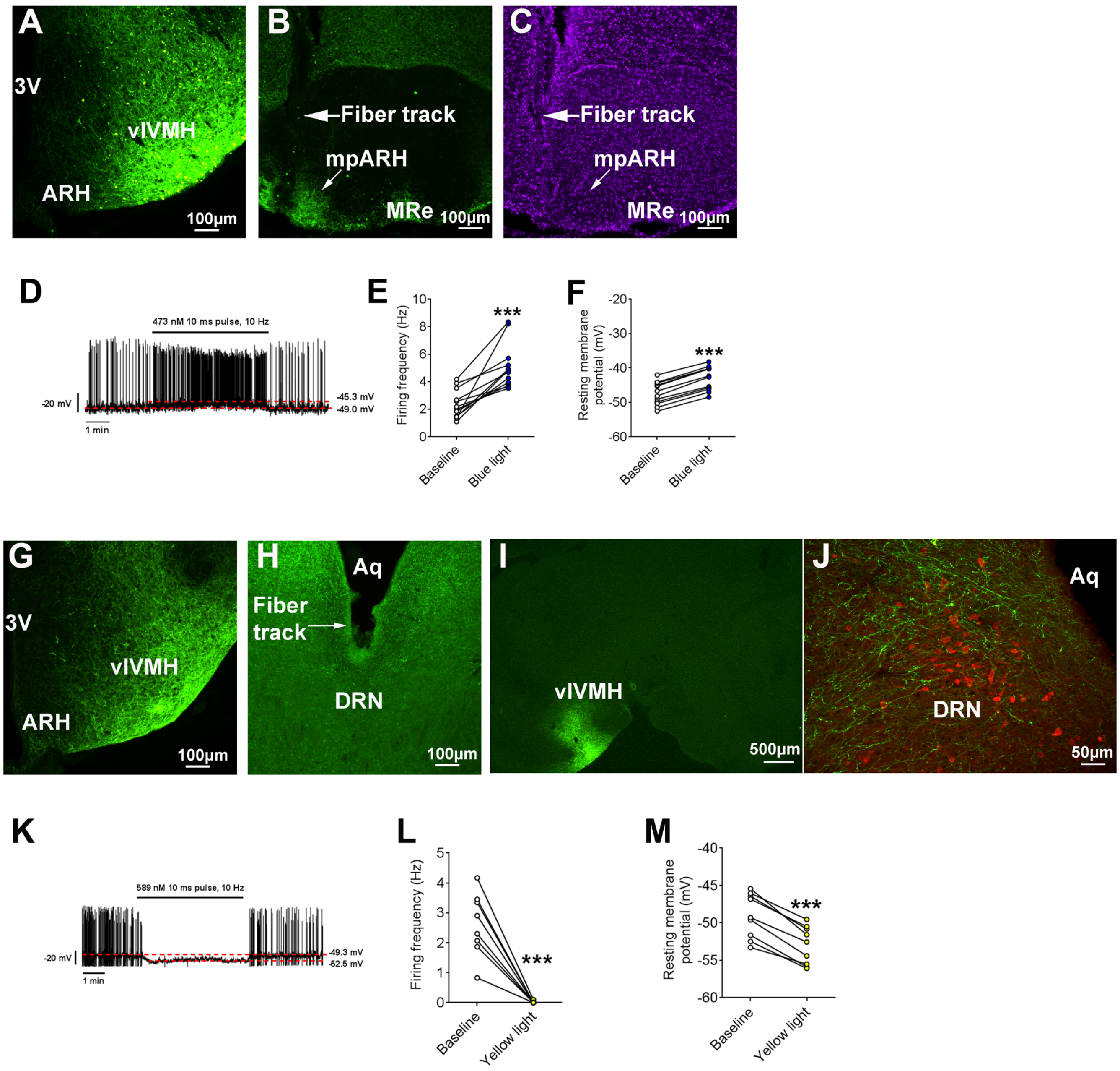

Supplementary Figure 5. Photostimulation/inhibition of ERα^{vlVMH}-originated circuits (related to Figure 5). (A) A representative fluorescent microscopic image showing expression of ChR2-EYFP in the vlVMH in female mice. The similar results were replicated in 5 mice. (B-C) Representative fluorescent microscopic images showing the optical fiber track in the mpARH with ChR2-EYFP-labeled fibers/terminals (B) or with a DAPI counter staining (C). The similar results were replicated in 5 mice. (D) Representative electrophysiological responses to blue light pulses in a female ChR2-EYFP-infected ERα^{vlVMH} neuron. (E and F) Firing frequency (E) and resting membrane potential (F) before and during blue light pulses in ChR2-EYFP-infected female ERα^{vlVMH} neurons. N=8 or 9 neurons. *** P=0.0001 in (E) and *** P<0.0001 in (F) in two-sided paired t-test. (G-H) Representative fluorescent microscopic images showing expression of eNpHR3.0-EYFP in the vlVMH (G) and the optical fiber track in the DRN (H) in coronal brain sections of female mice. Note that the EYFP-labelled fibers/terminals were not appreciable in the DRN in these coronal sections, thus we re-examined these fibers/terminals using sagittal section as described below. The similar results were replicated in 5 mice. (I-J) Representative fluorescent microscopic images showing expression of eNpHR3.0-EYFP in the vlVMH (I) and in the

DRN (J) in sagittal brain sections of female mice; 5-HT was counter-stained as a landmark of the DRN (red cells in J). The similar results were replicated in 3 mice. 3rd, 3rd ventricle; Aq, aqueduct; ARH, arcuate nucleus of hypothalamus; DRN, dorsal Raphe nuclei; mpARH, medioposterior arcuate nucleus of the hypothalamus; MRe, mammillary recess of the 3rd ventricle; vlVMH, ventrolateral subdivision of the ventromedial hypothalamic nucleus. (K) Representative electrophysiological responses to yellow light pulses in an eNpHR3.0-EYFP-infected female $ER\alpha^{vlVMH}$ neuron. (L and M) Firing frequency (L) and resting membrane potential (M) before and during yellow light pulses in eNpHR3.0-EYFP-infected female $ER\alpha^{vlVMH}$ neurons. N=8 or 9 neurons. *** P=0.0002 in (L) and *** P<0.0001 in (M) in two-sided paired t-test. Source data are provided as a Source Data Supplementary Figure 5.

Sample ID	# reads (post quality trim)	Unique reads (%)	Multi-mapping reads (%)	Unmapped reads (%)
GEneuron1	7412722	37.98	5.83	55.73
GEneuron4	4539153	54.31	9.27	35.54
GEneuron6	3037402	62.03	12.01	24.82
GEneuron8	3374059	66.17	9.52	23.24
GIneuron2	3593695	57.48	10.87	30.31
GIneuron3	54994	32.08	4.83	62.65
GIneuron5	4293928	54.62	10.66	33.79
GIneuron7	3308099	59.50	9.34	30.22

Supplementary Table 1 (related to Figures 2-3). Statistics of Patch-seq data and STAR alignment results. One sample (GIneuron 3) had much smaller number of short reads and was removed in analysis.

Sample ID	# total fragments	Assigned fragments (%)
GEneuron1	4055924	58.7
GEneuron4	3576101	55.7
GEneuron6	2917922	53.8
GEneuron8	3166915	60.3
GIneuron2	3214464	52.8
GIneuron3	25377	51.3
GIneuron5	3639064	52.8
GIneuron7	1646684	57.5

Supplementary Table 2 (related to Figure 2-3). Statistics of featureCounts analysis of Patch-seq data.

Gene/Protein	Primer Names	Sequence
Abcc8	Abcc8 F	GAGATCGCTGAGGGTATCCTG
	Abcc8 R	TTGGGGAAGTTAGAGGTCTCAAT
Ano4	Ano4 F	GGAGATGGTTACCTAAGAAGCCA
	Ano4 R	AGCGGTTCAGACCGATTTTATTC
Cyclophilin	CYCLO-QF	TGGAGAGCACCAAGACAGACA
	CYCLO-QR	TGCCGGAGTCGACAATGAT

Supplementary Table 3 (related to Figure 2-3). Sequences of qPCR primers.
