# Supplementary material for: Estrogen receptor-α expressing neurons in the ventrolateral VMH regulate glucose balance
Source: Nat Commun. 2020 May 1;11:2165. doi: 10.1038/s41467-020-15982-7 (PMC7195451; doi:10.1038/s41467-020-15982-7)
Supplement: Supplementary file 2 — Description of Additional Supplementary Items [file 41467_2020_15982_MOESM2_ESM.pdf]

Description of Additional Supplementary Files

File Name: Supplementary Data 1
Description: (related to Figures 2-3). List of 372 genes [P<0.05 and |log2(fold change)| >2] with different abundance in GE-ERαvlVMH neurons vs. GI-ERαvlVMH neurons from female mice.

File Name: Supplementary Data 2
Description: (related to Figures 2-3). Gene sets enriched in the 372 genes (FDR <0.2). WebGestalt (version 2019) was used. Only those gene sets with size 5-2000 were considered. FDR: adjusted P value (two-sided test) by Benjamini-Horchberg procedure for multiple test correction.

File Name: Supplementary Movie 1
Description: (related to Figure 5). Esr1-Cre female mice received stereotaxic injections of the AAV-EF1α-DIO-hChR2(H134R)-EYFP into the vlVMH, and an optic fiber was implanted to target at the DRN. Video recording behaviors of one mouse receiving blue light photostimulation (front) and another mouse receiving yellow light as a control (back). No aggressive, social and sexual behaviors were observed in these singly-housed mice. The same experiment was repeated in 5 female mice with the same outcome.
