# Supplementary material for: Estrogen receptor-α expressing neurons in the ventrolateral VMH regulate glucose balance
Source: Nat Commun. 2020 May 1;11:2165. doi: 10.1038/s41467-020-15982-7 (PMC7195451; doi:10.1038/s41467-020-15982-7)
Supplement: Supplementary file 6 — Reporting summary [file 41467_2020_15982_MOESM6_ESM.pdf]

nature research

Corresponding author(s): Yong Xu

Last updated by author(s): 03/16/2020

Reporting Summary

Nature Research wishes to improve the reproducibility of the work that we publish. This form provides structure for consistency and transparency in reporting. For further information on Nature Research policies, seeAuthors & Referees and theEditorial Policy Checklist .

Statistics

For all statistical analyses, confirm that the following items are present in the figure legend, table legend, main text, or Methods section.

n/a	Confirmed	
☐	☒	The exact sample size (n) for each experimental group/condition, given as a discrete number and unit of measurement
☐	☒	A statement on whether measurements were taken from distinct samples or whether the same sample was measured repeatedly
☐	☒	The statistical test(s) used AND whether they are one- or two-sided<br>*Only common tests should be described solely by name; describe more complex techniques in the Methods section.*
☐	☒	A description of all covariates tested
☐	☒	A description of any assumptions or corrections, such as tests of normality and adjustment for multiple comparisons
☐	☒	A full description of the statistical parameters including central tendency (e.g. means) or other basic estimates (e.g. regression coefficient) AND variation (e.g. standard deviation) or associated estimates of uncertainty (e.g. confidence intervals)
☐	☒	For null hypothesis testing, the test statistic (e.g. F, t, r) with confidence intervals, effect sizes, degrees of freedom and P value noted<br>*Give P values as exact values whenever suitable.*
☒	☐	For Bayesian analysis, information on the choice of priors and Markov chain Monte Carlo settings
☒	☐	For hierarchical and complex designs, identification of the appropriate level for tests and full reporting of outcomes
☒	☐	Estimates of effect sizes (e.g. Cohen's d, Pearson's r), indicating how they were calculated

Our web collection on statistics for biologists contains articles on many of the points above.

Software and code

Policy information about availability of computer code

Data collection	Eletrophysiological data were sampled using Digidata 1440A and analyzed offline with pClamp 10.3 software. Microscopic images were analyzed using the Leica MM AF Acquisition and Analysis (Cat#: 11640901). Fiber photometry data were collected using Synapse 2.0 software. Real-time PCR data were collected using Bio-Rad CFX Manager (3.1).
Data analysis	RNA-seq raw data files were trimmed using TrimGalore (version 0.4.1) and aligned against the mouse reference genome assembly (GRCm38.p6) using the STAR aligner (version 2.5.3a). Gene set enrichment analysis was performed using the online tool WebGestalt (version 2019).

For manuscripts utilizing custom algorithms or software that are central to the research but not yet described in published literature, software must be made available to editors/reviewers. We strongly encourage code deposition in a community repository (e.g. GitHub). See the Nature Research guidelines for submitting code & software for further information.

Data

Policy information about availability of data

All manuscripts must include a data availability statement. This statement should provide the following information, where applicable:

- Accession codes, unique identifiers, or web links for publicly available datasets
- A list of figures that have associated raw data
- A description of any restrictions on data availability

Raw data of Patch-seq study has been deposited in NCBI ([GEO]GSE146543). Further information and requests for raw data, resources and reagents should be directed to and will be fulfilled by Yong Xu (yongx@bcm.edu).

Field-specific reporting

Please select the one below that is the best fit for your research. If you are not sure, read the appropriate sections before making your selection.

- [x] Life sciences
- [ ] Behavioural & social sciences
- [ ] Ecological, evolutionary & environmental sciences

For a reference copy of the document with all sections, see nature.com/documents/nr-reporting-summary-flat.pdf

Life sciences study design

All studies must disclose on these points even when the disclosure is negative.

Sample size	The sample size was predetermined based on pilot studies and GraphPad StatMate analyses to ensure sufficient power to reach statistical significance (P<0.05).
Data exclusions	One RNA-seq sample ("GIneuron3") was removed from the following analysis due to low sequence read counts; 3 blood samples for glucagon measures were removed because the readouts were below the detecting threshold likely due to hemolysis. These criteria for exclusion were pre-established prior to the data collection.
Replication	All studies were replicated in multiple biological samples. In most experiments, the number of replications were indicated as N values in each figure. For microscopic images, similar results were repeated at least 3 times and the information was provided in each figure legend.
Randomization	Study animals were randomly divided into different treatment groups.
Blinding	For electrophysiology recordings, the investigator was not blinded for animals' genotypes, but he was blinded with the treatments (e.g. virus injections) the animals were subjected to. For the measurement of glucose, investigators were blinded for animal's genotypes. For Patch-seq study and real-time RT-qPCR study, the investigator was blinded with the nature of neurons (GE or GI). For fiber photometry study, investigators were blinded for the location of virus injections. For WGA tracing study and the ChR2-assistant circuit mapping, the investigator was not blinded because the treatment was the same cross the animals, and the investigator need to explore the target sites.

Reporting for specific materials, systems and methods

We require information from authors about some types of materials, experimental systems and methods used in many studies. Here, indicate whether each material, system or method listed is relevant to your study. If you are not sure if a list item applies to your research, read the appropriate section before selecting a response.

Materials & experimental systems

n/a	Involved in the study	
	x	Antibodies
x		Eukaryotic cell lines
x		Palaeontology
	x	Animals and other organisms
x		Human research participants
x		Clinical data

Methods

n/a	Involved in the study	
x		ChIP-seq
x		Flow cytometry
x		MRI-based neuroimaging

Antibodies

Antibodies used	Goat anti-WGA antibody (VectorLabs, AS-2024) and biotinylated donkey anti-goat secondary antibody (Jackson ImmunoResearch, 705-065-003)
Validation	The WGA antibody has been validated in my lab using mouse brain tissues without infections of WGA-expressing vectors.

Animals and other organisms

Policy information about studies involving animals; ARRIVE guidelines recommended for reporting animal research

Laboratory animals	Several transgenic mouse lines including ERα-ZsGreen, ERα-ZsGreen/Rosa26-TOMATO, and Esr1-Cre were maintained on a C57BL6/J background. Esr1-Cre mice were purchased from Jackson Laboratory (#017911) which express Cre recombinase selectively in ERα-expressing neurons, including those in the vlVMH. In addition, some C57Bl6J mice were purchased from the mouse facility of Baylor College of Medicine. Mice were housed in a temperature-controlled environment at 22˚C-24˚C using a 12 hr light/12 hr dark cycle. The mice were fed standard chow (6.5% fat, #2920, Harlan-Teklad, Madison, WI). Water was provided ad libitum. Both male and female mice (aging from 12-20 weeks) were used.
Wild animals	The study did not involve wild animals.

Field-collected samples	The study did not involve samples collected from the field.
Ethics oversight	Care of all animals and procedures were approved by the Baylor College of Medicine Institutional Animal Care and Use Committee.

Note that full information on the approval of the study protocol must also be provided in the manuscript.
